# Interventions to enhance healthcare utilisation among pregnant women to reduce maternal mortality in low- and middle-income countries: a review of systematic reviews

**DOI:** 10.1186/s12889-023-16558-y

**Published:** 2023-09-06

**Authors:** Themba Mzembe, Victor Chikwapulo, Tony Mwenda Kamninga, Ruth Vellemu, Sahra Mohamed, Lomuthando Nthakomwa, Chimwemwe Chifungo, Kerri Wazny, Kelvin Musau, Leila Abdullahi, Maame Peterson, Nyovani Madise, Michael G. Chipeta

**Affiliations:** 1 African Institute for Development Policy (AFIDEP), Lilongwe, Malawi; 2 Equity and Social Policy, ODI, London, UK; 3 The Children’s Investment Fund Foundation, London, UK; 4 The Children’s Investment Fund Foundation, Nairobi, Kenya

**Keywords:** Maternal mortality, Low and middle income, Antenatal care, Postnatal care

## Abstract

**Background:**

Maternal mortality in low- and middle-income countries (LMIC) has reduced considerably over the past three decades, but it remains high. Effective interventions are available, but their uptake and coverage remain low. We reviewed and synthesised evidence from systematic reviews on interventions to increase healthcare services utilisation to reduce maternal mortality in LMICs.

**Methods:**

We searched Medline PubMed and Cochrane Library databases for systematic reviews published between January 2014 and December 2021, investigating interventions to increase healthcare services uptake among pregnant women in LMICs. We used the AMSTAR tool (A Measurement Tool to Assess Systematic Reviews) to assess the methodological quality of the included reviews. We extracted data on the interventions and their effects and grouped them into broad groups based on the outcomes reported in each systematic review.

**Results:**

We retrieved 4,022 articles. After removing duplicates and screening, we included 14 systematic reviews.

Male-partner interventions were effective in increasing skilled birth attendance (SBA) postnatal visits and maternal antiretroviral (ART) uptake for HIV-positive pregnant women. However, there was no evidence of their effectiveness on increased early ANC initiation or adequate ANC visits.

Mobile health interventions were effective in increasing adequate ANC visits, SBA, facility-based service utilisation, early ANC initiation, and adherence to nutritional supplements.

Incentive-based interventions, particularly financial incentives, were effective in increasing the number of ANC visits but not postnatal visits.

Facility-based interventions were effective in increasing postnatal visits, maternal ART initiation and uptake, immunisation uptake and follow-up ANC visits. None of the reviews assessed their impact on SBA or adequate ANC visits.

Community-based interventions were effective in increasing SBA, ANC service utilisation, ART initiation and uptake, and nutritional supplements and immunisation uptake.

**Conclusion:**

Our findings show that the different interventions effectively improved different outcomes on the maternal healthcare continuum. Implementing these interventions in combination has the potential to enhance healthcare service uptake further.

**Supplementary Information:**

The online version contains supplementary material available at 10.1186/s12889-023-16558-y.

## Background

With over 295,000 women and girls dying yearly due to pregnancy-related problems, maternal mortality remains a critical global healthcare challenge [1]. Low-and middle-income countries (LMICs) are disproportionately affected as over 94% of all maternal deaths occur in these countries, with sub-Saharan Africa (SSA) alone contributing about 66% of all maternal deaths. The estimated lifetime risk of maternal death in SSA in 2017 was 1 in 37, compared to just 1 in 7,800 in developed countries like Australia and New Zealand [1].

Between 1990 and 2017, the global maternal mortality ratio (MMR) decreased by 44%, from 385 to 216 maternal deaths per 100,000 live births [2]. However, this fell short of the Millennium Development Goal target of reducing the global MMR by 75% in 2015 compared to 1990 [3]. Despite this remarkable progress, the number of pregnant women dying from preventable causes remains unacceptably high [1].

Globally, the leading causes of maternal mortality include high blood pressure during pregnancy, excessive bleeding (mainly after delivery), sepsis, complications from childbirth and unsafe abortions, and infection with diseases such as acquired immunodeficiency syndromes (AIDS) and malaria [1]. Improved healthcare service utilisation can effectively mitigate these complications among pregnant women during pregnancy (antenatal care; ANC), intrapartum (during labour and delivery), and postpartum (after delivery) periods [4].

Early and adequate ANC visits can significantly reduce maternal mortality and morbidity through timely identification and management of pregnancy-related complications. The 2016 World Health Organization (WHO) guidelines recommend at least eight visits during pregnancy, with the first occurring in the first trimester (less than 12 weeks gestation), as this ensures a healthy pregnancy, an effective transition to labour and delivery, and a positive motherhood experience [5]. Timely and adequate ANC visits have been associated with increased usage of emergency obstetric care, institutional delivery, and positive pregnancy outcomes [6, 7].

Despite the enormous benefits of early and adequate ANC visits on pregnancy outcomes, the uptake of services among pregnant women in LMICs remains low. Many pregnant women continue to present to care late. A systematic global analysis of trends of early ANC visits between 1990 and 2013 showed that the estimated coverage of early ANC visits in low-income countries in 2013 was only 24% compared to 82% in high-income countries [8]. While the estimated global ANC coverage (defined as at least one ANC visit) had increased to 86% in 2019, only 52% and 46% of pregnant women made at least four ANC visits in sub-Saharan Africa and south Asia, respectively [9].

Within LMICs, low educational attainment, poor socioeconomic status, rural residence, and increased distance to health facilities are among the leading factors associated with late presentation to healthcare services [8, 10–13]. Pregnant women in hard-to-reach rural areas are more likely to die from pregnancy-related complications than those in urban areas, as most facilities in rural areas are not well equipped, and pathways to referral facilities are costly and complicated [14].

The emergence of the coronavirus disease (COVID-19) pandemic has further compounded challenges on the already struggling health systems in LMICs by causing disruptions in delivery of healthcare services. Limited financial and human resources intended for improving services like maternal healthcare have to be diverted towards the imminent need to contain the spread of the severe acute respiratory syndrome coronavirus 2 (SARS-CoV-2) infection [15, 16]. Consequently, adversely affecting the progress made by countries in reducing the maternal mortality rate (MMR) to the target of less than 70 maternal deaths per 100,000 live births by 2030 [17]. To accelerate progress towards this goal, simple, cost-effective interventions that can increase healthcare utilisation among pregnant women must be implemented or scaled up in LMICs. Several systematic reviews have identified, evaluated, and summarised the findings from relevant individual studies of effective interventions [18–22]. In an effort to inform future research and provide policy makers with timely evidence for evidence-informed policy formulation, we aimed to synthesize evidence from systematic reviews on interventions aimed at improving healthcare service utilisation among pregnant women in LMICs to reduce maternal mortality.

## Methods

We used the methodology for rapid reviews provided in the interim guidance on rapid reviews from the Cochrane Rapid Reviews Methods Group [23] as stipulated in the protocol (Prospero protocol number CRD42021291467) for this review. We used the tool as it provides a standardized approach for conducting reviews that are rigorous, transparent, and useful for synthesizing timely evidence to inform decision-making quickly and efficiently.

### Inclusion and exclusion criteria

We used the following PICOST (population, intervention, comparator, outcome, setting and time) matrix to include or exclude studies in the review:

1. *Population*: Women of reproductive age (15–49 years).
2. *Intervention*: Any intervention (including policy changes) that may lead to increased healthcare service utilisation among pregnant women.
3. *Study setting*: Low-and-middle-income countries. We used the World Bank’s classification of countries by income released on 1st July 2021, available at: https://datahelpdesk.worldbank.org/knowledgebase/articles/906519-world-bank-country-and-lending-groups.
4. *Comparator*: we had no pre-specified comparator. We reported the comparison groups as reported in the included reviews.
5. *Outcome*: The primary outcome was increased uptake of healthcare services as a percentage following the intervention or any measure of effect (odds ratio, risk ratio) for improved healthcare utilisation in the treatment group compared to the control group.
  Some specific outcomes were:
  1. Increased early ANC initiation (less than 12 weeks gestation or in first trimester).
  2. Increased adequate ANC visits (at least four visits).
  3. Increased facility deliveries.
  4. Increased skilled attendance at delivery (by doctor, midwife, trained nurse).
  5. Increased postnatal follow-up visits.
  6. Increased essential vaccine uptake for pregnant women.
  7. Increased nutritional supplement uptake.
  8. Increased uptake of treatment for infections (for example, HIV infection).

6. *Study design*: Systematic reviews.

We included published systematic reviews assessing interventions for improving healthcare service uptake among pregnant women in LMICs, irrespective of the designs of the included studies in the reviews. We included both Cochrane and non-Cochrane Reviews, provided they had used a systematic approach to identify the included studies. We included systematic reviews with and without meta-analyses but excluded meta-analyses without systematic reviews. We included systematic reviews with at least one study conducted in an LMIC setting.

### Time

We included systematic reviews published between January 2014 to December 2021 as we were interested in identifying interventions which would be more applicable in accelerating the achievement of the sustainable development goal (SDG) to reduce the global MMR to less than 70 maternal deaths by 2030 [17].

### Search methods for identification of reviews

We used keywords and Medical Subject Headings (MESH) to perform electronic searches in Medline PubMed and the Cochrane Library databases using the following search terms: “maternal health”, “maternal mortality”, “interventions for reducing maternal mortality”, and “Low- and middle-income countries”. We searched the literature for synonyms of each search term to develop a comprehensive search strategy. Appendices 1 and 2 provide the search strategies for each of the databases. Additionally, we screened the reference lists of all the included reviews for other potentially eligible reviews.

### Data collection and analysis

#### Selection of reviews

We used a two-step screening process; in the first step, two review authors (TM and VC) independently screened the titles and abstracts of studies retrieved from the electronic databases. After that, we obtained full texts of eligible studies for further review and the final selection of eligible studies for inclusion in the review. In case of disagreements, we thoroughly examined the issues and consulted a third-party opinion (LA, MA, or LN) to resolve them.

### Data extraction and management

We developed a data extraction tool (Supplementary Material Appendix 3) which we piloted on five systematic reviews before the actual data extraction to enhance the consistency of the results. After verifications, we used the tool to develop a Microsoft Access (Microsoft Corporation, Redmond, Washington, United States) database to extract data from the included reviews. The form also guided specific data extraction and recording formats for uniformity.

Where possible, we extracted the following data from the included systematic reviews:

1. Characteristics of the included systematic reviews: date of search; the number of studies included and the number of participants in each study; review objective(s); type of participants; setting (countries); interventions; comparisons; relevant outcomes with definition and information for any adjustments.
2. GRADE (Grading of Recommendations, Assessment, Development and Evaluations) assessment of relevant outcomes.
3. Risk of bias (RoB) assessment in the included systematic reviews: methods used; domains assessed; judgements.
4. Characteristics of interventions: description of the intervention; the form of application frequency; start and duration of intervention; adherence to the intervention.
5. Results of included reviews: comparison group; outcome; numbers of studies and participants; results (from meta-analysis or narrative description).

Where the systematic reviews included studies from both LMICs and high-income countries, we extracted data only from studies conducted in LMICs. However, where the results were combined in a meta-analysis, we extracted the combined result from the meta-analysis. Where any information from the reviews was unclear or missing, we accessed the published papers of the individual studies included in the reviews.

### Quality assessments

#### Assessment of methodological quality of included reviews

We used the AMSTAR tool (A Measurement Tool to Assess systematic Reviews [24, 25]) as provided in Supplementary Material Appendix 4 to assess the methodological quality of the included reviews.

#### Quality of the evidence in included reviews

We extracted information on the RoB methods and ratings used in the included systematic reviews. In addition, where provided in the reviews, we extracted GRADE ratings [26] for the outcomes of interest for this review to assess the certainty of the evidence.

### Data synthesis

We grouped the interventions into broad groups based on the outcomes measured for each intervention in each systematic review.

We tabulated PICOS (population, intervention, control, outcome and setting) elements at the review level. Results tables included effect estimates, 95% confidence intervals (CIs), and measures of heterogeneity/RoB, as appropriate.

The choice of effect estimates for summary and tabulation depended on the outcomes reported in the selected reviews. Where possible, we standardised the results reported if outcomes were expressed differently between studies. We standardised the effect estimates to risk ratios (RRs) or odds ratios (ORs) for dichotomous outcomes and mean differences (MDs) or standardised mean differences (SMDs) for continuous outcomes.

## Results

### Description of included systematic reviews

**Fig. 1:**
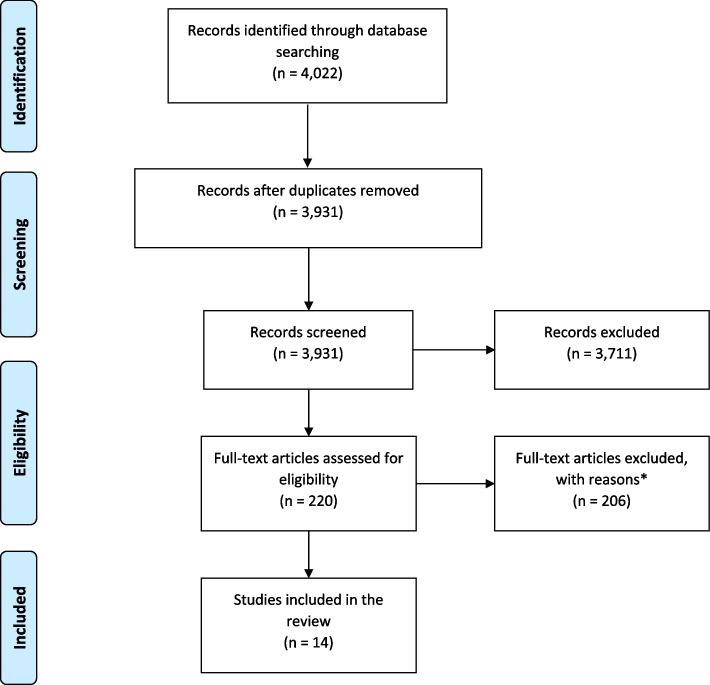
Flow diagram for the selection process of studies identified and included in the review. *Reasons for exclusion:Preventing or managing pregnancy related complications (55), Favourable pregnancy outcomes and not improvement of healthcare service uptake (38), Preventing or managing infections during pregnancy (34), Treating or preventing anaemia or postpartum haemorrhage (27), Safe abortion or preventing pregnancy (22), Preventing or managing pre-eclampsia (18), Assessing safety or acceptability of an intervention not effect on increasing healthcare uptake (3), Assessing effect of different nutritional supplements and not increasing service uptake (2), Not looking at pregnant women (2), Sexual reproductive health and rights—not focussing on outcomes during pregnancy (2), Improving quality of care and not service uptake (1), Intervention in humanitarian setting (1), Assessing different antenatal care models (1)

We retrieved 4,022 systematic reviews (Fig. 1). After removing duplicates, title and abstract screening, and performing full-text critical appraisals, we included 14 systematic reviews.

Table 1 provides the details of the included systematic reviews. The systematic reviews were published from 2015 to 2021 and had between 3 to 47 studies. Four reviews (Mbuagbaw et al. [27], Seward et al. [28], Till et al. [29] and Wagnew et al. [30]) included intervention (RCT, cRCT and pre-post studies) studies only, while the rest had a combination of intervention and observation (cohort, cross-sectional and case–control) studies.

**Table 1:** Characteristics of included systematic reviews

First author; Year; Review aim	Intervention description	Intervention delivery	Study country (number of studies) and Study designs	Participants included	GRADE & RoB Method
**Lassi and Bhutta 2015 **[32]					
Assess the effectiveness of community-based intervention packages in reducing maternal and neonatal morbidity and mortality	Training of outreach workers, e.g., community/village health workers in maternal care during pregnancy, delivery and in the postpartum period; and routine new-born care	TBAs were trained for enhanced complication referrals, Improved accessibility to healthcare services Organized village women’s groups and held monthly meetings to address obstetric and perinatal problems	25 studies conducted in India (5 studies); Bangladesh (6 studies), Pakistan (3), Gambia (2 studies), Nepal (3 studies), Indonesia (1 study), Burkina Faso (1) study, Gambia (2 studies) and Bolivia (2 studies) clustered randomized controlled trials-12, quasi randomized-9, Pre–post studies with no control arm-4	Women of reproductive age group, particularly pregnant women at any period of gestation	**GRADE:** Not assessed **RoB tool:** An adaptation of the Quality assessment tool
**Lassi et al., 2015 **[40]					
Evaluate the delivery mechanisms which have been implemented over the years in different parts of the world to improve maternal and new-born health in conflict-affected areas	Community outreach services- Community Health Workers (CHW) training, labour room services provision for an internally displaced population Programme activities included: Raising community awareness, making the health facilities operational, transferring skills to the district health team, changing the health-related behaviour of the population Facility-based services: Through a healthcare centre, community sensitization to HIV, training of healthcare workers, voluntary counselling and HIV testing	Community-based services: Training CHWs as part of the Basic Package of Health Services Community-based services: CHWs, TBAs and maternal health workers were trained for eight months and allowed to work in the community for two years	3 studies conducted in Myanmar (1 study), Democratic Republic of the Congo (1 study), and Tanzania (1 study) Case Study-1, Pre-post Surveys-1 and Not clear study design-1	Community health worker Married women of reproductive age (15–45 years)	**GRADE:** Not assessed **RoB tool:** The Cochrane handbook for systematic reviews
**Geremew et al., 2020 **[39]					
Assess pooled evidence on the effect of antenatal care service utilization on postnatal care service utilization	ANC service utilization		14 studies were conducted in Rwanda (2 studies), Ethiopia (7 studies), Kenya (2 studies), South Sudan (1 study), Tanzania (1 study) and Zambia (1 study) Retrospective follow-up-1 and Cross-sectional-13	Women who use ANC and PNC	**GRADE:** Not assessed **RoB tool:** The Joana Briggs Institute (JBI) critical appraisal tools
**Geldsetzer et al*.*, 2016 **[36]					
Assess the evidence for innervations that aim to improve retention within the ART program, transitioning from PMTC to the general ART program and retention in the general ART program	Use of text messaging and/or phone calls	Two-way text messaging	7 studies conducted in Kenya (4 studies), Uganda (1 study), and Malawi (2 studies) Individually randomized studies-3, CRT-1, Cohort study-2 and Pre/post cohort study-1	Pregnant /postpartum women with HIV	**GRADE:** Assessed using the GRADE criteria **RoB tool:** Not available
**Brittain et al., 2021 **[41]					
Collate the available evidence on strategies to improve retention in antenatal and/or postpartum care among adolescents and young WLHIV and also a review of strategies to increase attendance at antenatal care (ANC) and/or facility delivery among pregnant adolescents, regardless of HIV status, to identify approaches that could be adapted for adolescents and young WLHIV	Integrated care during the postpartum period a lay counsellor-led combination intervention Mobile health technologies enhanced support Active follow-up and tracing Home visits Continuity of care with the same provider	Community and health facility	47 studies conducted in DRC (1 study), Malawi (4 studies), Kenya (6 studies), Nigeria (3 studies), Tanzania (5 studies), Zambia (2 studies), South Africa (8 studies), Mozambique (1 study), Uganda (3 studies), Lesotho (1 study), Zimbabwe (3 studies), India (1 study), Egypt (1 study), Australia (1 study), Canada (1 study), England (1 study) and USA (4 studies) RCT-11, Cluster RCT-10, Group RCT-1, Pilot RCT-3, Before-after cohort study-1, 3-arm cluster RC-1, Retrospective cohort-8, Matched cohort-1, Cohort-2, Prospective paired cohort-1, Retrospective intervention study-1, Stepped-wedge cluster RCT-2 and Pre-post intervention study-5	Adolescent and young women living with HIV	**GRADE:** Not assessed **RoB tool:** Not available
**Wagnew et al., 2018 **[30]					
Determine the effectiveness of short message services (SMS) on Focused Antenatal Care (FANC) visits and the attendance of skilled birth professionals in Low- and Middle-Income Countries (LMICs)	Interventions that use SMS or phone calls as reminders for a scheduled health appointment(s)	Texting or calling the participants as a reminder to attend the clinic	7 studies conducted in Thailand (1 study), Zanzibar (2 studies), Kenya (1 study), South Africa (1 study), Ethiopia (1 study) and India (1 study) RCT-7	Pregnant women	**GRADE:** Assessed using GRADE criteria **RoB tool:** The Cochrane Collaboration’s Tool
**Vrazo et al., 2018 **[38]					
Summarise the interventions that demonstrate statistically significant improvements in service uptake and retention of HIV-positive pregnant and breastfeeding women and their infants along the PMTCT cascade	Increase access to antenatal care (ANC) and ART services Using lay cadres quality improvement mHealth counselling		12 studies conducted in South Africa (3 studies), Malawi (2 studies), Kenya (3 studies), DRC (1 study), Zambia (1 study), and Cote d’Ivoire (1 study) Cluster RCT-2, Prospective cohort-5, RCT-1, Retrospective cohort-1 and Pre–post-comparison-2	HIV-positive pregnant women and HIV-exposed infants	**GRADE:** Not assessed **RoB tool:** An eight-item assessment tool
**Takah et al., 2017 **[33]					
Identify the approaches that are used in improving male partner involvement in the prevention of mother-to-child transmission (PMTCT) of HIV and their impact on the uptake of maternal antiretroviral therapy (ART) in sub-Saharan Africa (SSA)	Invitation letters to male partners to be involved in PMTCT Psychological counselling conducted by trained personnel, e.g., women to disclose their HIV status to their male partners Verbal encouragement through counselling to bring partners to the antenatal clinic	Invitation letters to male partners to be involved in PMTCT	17 studies conducted in South Africa (2 studies), Tanzania (2 studies), Nigeria (1 study), Kenya (3 studies), Mozambique (1 study), Uganda (2 studies), Rwanda (2 studies), Zambia (1 study) and Malawi (3 studies) RCT-4, Cohort-11, Serial cross-sectional-1	HIV-positive pregnant women and breastfeeding mothers with their male partners	**GRADE:** not assessed **RoB Tool:** Jadad Scale and Newcastle–Ottawa Scale
**Till et al., 2015 **[29]					
Determine whether incentives are an effective tool to increase the utilization of timely prenatal care among women	Household-level package communities: Eligible households receive vouchers for each pregnant woman, a child under age 3 or between ages 6–12 enrolled in school Service-level package communities received quality improvement teams to strengthen health centres and community-based nutrition programs Dual-package communities received both household-level and service-level interventions	Household/ community level	Four studies conducted in USA (3 studies), Mexico (1 study) and Honduras (1 study) Cluster RCT-2 and RCT-3	Pregnant women	**GRADE:** Not assessed **RoB Tool:** Cochrane Handbook for Systematic Reviews
**Tibingana-Ahimbisibwe et al*.*, 2016 **[37]					
Systematically review the effect of adolescent-specific interventions on reducing Preterm birth (PTB), low birth weight (LBW), perinatal death and increasing prenatal care attendance	Hospital-based comprehensive interdisciplinary, and adolescent-specific prenatal care programme		22 studies conducted in USA (15 studies), UK (2 studies), Canada (3 studies), Australia (1 study) and Egypt (1 study) RCT-3, Prospective cohort-4, Retrospective cohort -9, Case control-5 and natural experiment -1	Pregnant adolescents (10–19 years)	**GRADE:** Not assessed **RoB tool:** A validated tool from the Effective Public Health Practice Project
**Suandi et al., 2021 **[34]					
Investigate whether involving male partners in antenatal care improves healthcare utilisation	Male involvement in maternal and child health (MCH) that required men to play more responsible roles in MCH to ensure women’s and children’s well-being	Husband’s pregnancy knowledge and wife’s participation in household decision making The male partner was invited to an interactive group discussion; couple’s counselling during pregnancy and postnatal unhyphenated throughout couple’s counselling session	17 Studies conducted in Nepal (3 studies), India (2 studies), Indonesia (1 study), Myanmar (1 study), Bangladesh (1 study), Kenya (1 study), Malawi (1 study), Zambia (1 study), South Africa (1 study), Burkina Faso (1 study), Uganda (1 study) and Ethiopia (3 studies) RCT-5, non-randomised controlled trial-1, cluster RCT-1, Cohort-3 and cross-sectional surveys-6	Women of reproductive age, although some were recruited from antenatal clinics, while others participated in surveys after pregnancy and delivery	**GRADE:** not assessed **RoB Tool: **Downs and Black Checklist
**Sondaal et al*.*, 2016 **[31]					
Assess the effect of mobile Health interventions that support pregnant women during the antenatal, birth and postnatal period in LMIC	Unidirectional text (and voice) messaging Direct two-way communication Both unidirectional and direct two-way communication Multidirectional text messaging Unidirectional telephone counselling		27 studies implemented in multiple countries: Afghanistan (1 study), Argentina (1 study), Bangladesh (2), Burkina Faso (1 study), China (1 study), Ghana (1 study), India (2 studies), Kenya (1 study), Lebanon (1 study), Malawi (1 study), Malaysia (1 study), Nigeria (1 study), Puerto Rico (1 study), Serbia (1 study), Sierra Leone (1 study), South Africa (4 studies), Tanzania (2 studies) and Thailand (3 studies), Iran (1 study) Intervention studies-12 and descriptive studies-15	Pregnant women	**GRADE:** Not assessed **RoB tool:** an adaptation to the Cochrane Collaboration’s tool
**Seward et al., 2017 **[28]					
Examine the effect of women's groups on key antenatal, delivery, and postnatal behaviours to understand pathways to mortality reduction	Women’s groups meeting regularly with the help of the female facilitator		7 studies implemented in Bangladesh (2 studies), Malawi (1 study), Nepal (1 study) and India (3 studies) RCTs-7	Women aged 15–49 years	**GRADE:** Not assessed **RoB tool:** Not available
**Saronga et al., 2019 **[35]					
Evaluate the effectiveness of mHealth interventions on improving dietary/nutrients intake of pregnant women in LMIC	Text messaging voice messages delivered via mobile phone combination of mobile phone calls and text messaging	Sending information via text or voice message, or phone call	4 studies were implemented: India (2 studies), Indonesia (1 study) and Kenya (1 study) RCT-3 and Pre-post study-1	Pregnant women	**GRADE:** Not assessed **RoB Tool:** Academy of Nutrition and Dietetics Tool

*ANC* Antenatal care, *ART* Antiretroviral therapy, *GRADE* Grading of Recommendations, Assessment, Development and Evaluations, *LMIC* Low- and middle-income country, *RCT* Randomized controlled trial, *RoB* Risk of bias, *PMTCT* Prevention of mother-to-child transmission, *PNC* Postnatal care, *TBA* Traditional birth attendant

The systematic reviews predominantly included studies conducted in SSA and South and South East Asia. One review by Sondaal et al. [31] also included one study conducted in Eastern Europe. Additionally, two reviews (Sondaal et al. [31] and Till et al. [29]) each included one study, while one review by Lassi and Bhutta [32] included two studies from Latin America.

In terms of interventions, two systematic reviews (Takah et al. [33] and Saundi et al. [34]) assessed the effect of interventions involving male partners. Four systematic reviews (Sondaal et al. [31], Wagnew et al. [30], Saronga et al. [35] and Geldsetzer et al. [36]) assessed the effect of mobile health interventions. One review by Till et al. [29] reported the effect of incentive-based interventions. Three reviews (Tibingana-Ahimbisibwe et al. [37], Vrazo et al. [38]and Geremew et al. [39]) assessed the effect of health systems facility-based interventions. Four reviews (Lassi et al. [40], Lassi and Bhutta [32], Seward et al. [28], and Vrazo et al. [38]) reported the effect of health systems community-based interventions, and one review by Brittain et al. [41] reported the effect of composite health-systems interventions on the uptake of services by pregnant women.

### The methodological quality of included reviews

#### Quality of included reviews

We assessed the methodological quality of the included systematic reviews using the AMSTAR tool (Supplementary Material Appendix 4). Table 2 shows the score ratings for each systematic review. Overall, the included systematic reviews were of high quality. The average score was 83.1% (9.1 of the 11 points in the AMSTAR tool). Four systematic reviews scored 90.9% (10/11), and the lowest review scored 63.6% (7/11), with the rest scoring 81.8% (9/11). The main reasons for failing to score full marks were failure to include a list of excluded studies with reasons (11/14 reviews) and not assessing the likelihood of publication bias (12/14 reviews).

**Table 2:** AMSTAR ratings for the included systematic reviews

Author, Year	1^a^	2^b^	3^c^	4^d^	5^e^	6^f^	7^g^	8^h^	9^i^	10^j^	11^k^	Total Score (%)
Brittain et al., 2021 [41]	YES	YES	YES	YES	NO	YES	NO	NO	YES	NO	YES	**7 (63.6)**
Geldsetzer et al., 2016 [36]	YES	YES	YES	YES	NO	YES	YES	YES	YES	NO	YES	**9 (81.8)**
Geremew et al., 2020 [39]	YES	YES	YES	YES	NO	YES	YES	YES	YES	YES	YES	**10 (90.9)**
Lassi et al., 2015 [40]	YES	YES	YES	YES	NO	YES	YES	YES	YES	NO	YES	**9 (81.8)**
Lassiet and Bhutta, 2015 [32]	YES	YES	YES	YES	YES	YES	YES	YES	YES	NO	YES	**10 (90.9)**
Saronga et al., 2019 [35]	YES	YES	YES	YES	NO	YES	YES	YES	YES	NO	YES	**9 (81.8)**
Seward et al., 2017 [28]	YES	YES	YES	YES	NO	YES	YES	YES	YES	NO	YES	**9 (81.8)**
Sondaal et al., 2016 [31]	YES	YES	YES	YES	NO	YES	YES	YES	YES	NO	YES	**9 (81.8)**
Suandi et al., 2021 [34]	YES	YES	YES	YES	YES	NO	YES	YES	YES	NO	YES	**9 (81.8)**
Takah et al., 2017 [33]	YES	YES	YES	YES	YES	NO	YES	YES	YES	YES	YES	**10 (90.9)**
Tibingana-Ahimbisibwe et al., 2015 [37]	YES	YES	YES	YES	NO	YES	YES	YES	YES	NO	YES	**9 (81.8)**
Till et al., 2015 [29]	YES	YES	YES	YES	YES	YES	YES	YES	YES	NO	YES	**10 (90.9)**
Vrazo et al., 2018 [38]	YES	YES	YES	YES	NO	YES	YES	YES	YES	NO	YES	**9 (81.8)**
Wagnew et al., 2018 [30]	YES	YES	YES	YES	NO	YES	YES	YES	YES	NO	YES	**9 (81.8)**

1^a^: A priori design provided

2^b^: Duplicate study selection and data extraction

3^c^: Comprehensive literature search performed

4^d^: Status of publication used as an inclusion criterion

5^e^: List of studies (included and excluded) provided

6^f^: Characteristics of included studies provided

7^g^: Quality of included studies assessed and documented

8^h^: Quality of included studies used appropriately in formulating conclusions

9^i^: Appropriate methods used to combine the findings of the studies

10^j^: Likelihood of publication bias assessed

11^k^: Conflict of interest stated

### Quality of the evidence in the included reviews

In this section, we provide the RoB assessment of the included studies and the GRADE assessment of the certainty of the evidence in the included systematic reviews.

#### Male-partner involvement interventions

Two systematic reviews (Takah et al. [33] and Saundi et al. [34]) reported the effect of interventions involving male partners on increasing the uptake of healthcare services among pregnant women. Both systematic reviews did not perform GRADE assessments for the certainty of evidence. Takah et al. [33] used the Jadad Scale Assessment Tool [42], developed by the Joana Briggs Institute (JBI) (formerly known as the Oxford Pain Research Group),to assess the quality of randomised studies and the Newcastle–Ottawa Scale (available at: http://www.ohri.ca/programs/clinical_epidemiology/oxford.asp) to evaluate the quality of non-randomised studies included in the review. The authors reported that the randomised studies included in the review had a moderate RoB, while the non-randomised studies had a low to moderate RoB.

Suandi et al. [34] used the Downs and Black Checklist [43] to assess the quality of the included studies as the studies had varied designs. The included studies had a moderate to low RoB, with an average score of 53.8% (14/26). Based on the checklist, the included studies had the lowest score on the external validity criteria (average score: 22%).

#### Mobile health interventions

Four reviews (Sondaal et al. [31], Wagnew et al. [30], Saronga et al. [35] and Geldsetzer et al. [36]) reported the effect of mobile health interventions on the uptake of services among pregnant women. Sondaal et al. [31] and Saronga et al. [35] did not perform GRADE assessments for the certainty of evidence. Sondaal et al. [31] used an adapted Cochrane Collaboration tool for assessing RoB in randomised trials [44] to assess the quality of the included studies. The included studies were rated as having low RoB in terms of participant selection, completeness of data, clear outcome definition and assessment of the effect of confounders. The primary source of potential bias was measurement error for the outcomes and exposures.

Saronga et al. [35] used the Academy of Nutrition and Dietetics Tool for Primary Research [45] to assess the quality of the included studies. The review included four studies, of which two had positive quality, and the other two had neutral quality.

Wagnew et al. [30] used the Cochrane Collaboration’s Tool Assessing the quality of controlled clinical trials [46] to assess the quality of the included studies and the GRADE criteria [26] to evaluate the quality of evidence. Overall, the included studies had a low RoB but lacked clarity regarding assessment of blinding for outcome assessment. The evidence from the review was of moderate quality. Geldsetzer et al. [36] used the GRADE criteria [26] to assess the quality, and evaluate the quality of evidence from each study included in the review. The authors rated the evidence on the outcome for “attendance at PMTCT or postnatal clinic after delivery” as moderate quality. In contrast, the evidence on the outcome for retention on ART care at 12 months postpartum was of very low-quality (Table 3).

**Table 3:** Mobile health interventions

Review, year	Interventions and Comparisons	Outcomes	Number of studies (number of participants)	Results	GRADE or Risk of Bias Assessment
Sondaal et al., 2016 [31]	Pregnant women who got a phone call and text message reminder for next visit and educational messages vs routine care	Adequate ANC visits (≥ 4 visits)	1 (*n* = 2,637)	OR: 2.39, 95% CI: 1.03 –5.55 (Lund et al.)	Low risk of bias
		Skilled attendance at delivery	1 (*n* = 2,637)	OR: 5.73, 95% CI: 1.51 –21.81 (Lund et al.)	
		Increased facility-based service utilization (**Three** studies reported this outcome for this intervention)	(*n* = 8,110)	**- Study 1, Jalloh-Vos et al*.*:** Intervention showed a significant positive net effect but did not provide the effect estimate	
			(*n* = 3,230)	- **Study 2, Oyeyemi et al*.*:** Significantly higher in intervention areas than the control area (43.4% vs 36.7%, *p* = 0.0001), specifically primary healthcare facilities (54.5% vs 30.6%, *p* = 0.001)	
		Early ANC initiation and postnatal checks	(*n* = 6,479)	- **Study 3, Watkins et al*.*:** Increase in women attending ANC within the first trimester and a marginally statistically significant increase in postnatal check-ups within two days after birth. But did not provide an effect estimate	
	Pre and post-project evaluation of the same communities of text and phone call reminders: for ANC visits	Timely ANC visits	1 (*n* = 280)	OR: 2.97, 95% CI: 1.60—5.54 (43.79% before vs 58.68% after intervention) Kaewkungwal et al	
Wagnew, 2018 [30]	Text message reminders for next clinic visits vs routine services without text message reminders	Skilled attendance at delivery	3 (*n* = 3,282)	skilled birth attendance (OR = 1.82 (95% CI: 1.33, 2.49)	Moderate level of quality
		Adequate ANC visits	3 (*n *= 3,345)	Focused ANC visits (OR = 2.74 (95% CI: 1.41, 5.32)	
Saronga, 2019 [35]	Text or voice messages, and phone call reminders, plus giving support and advice vs routine care	Adherence to iron supplements	(*n* = 74)	Before intervention: 76.3% vs after intervention: 71.1% (Anitasari et al.)	Neutral quality
		Receiving vitamin supplements	(*n* = 397)	Intervention 39.8% vs control 23.8%, p < 0.001 (Fedha et al.)	Positive quality
		Receiving prophylactic iron and calcium tablets for ≥ 3 months	(*n* = 400)	Intervention: 81% vs control group: 69% (Bangal et al.)	Neutral quality
		Receiving iron supplements	(*n* = 397)	Intervention group 91.6%; control group 87.4%, *p* = 0.170 (Fedha et al.)	Positive quality
		Receiving dietary counselling	(*n* = 397)	Intervention: 95% vs control group 89.3%, *p* = 0.027 (Fedha et al.)	Positive quality
		Adequate ANC visits (**Two** studies reported this outcome for this intervention)	(*n* = 400)	Intervention: 57.5% vs control group: 23.5%, *p* < 0.001 (Bangal et al.)	Neutral quality
			(*n* = 397)	Intervention: 96.4% vs control group 90.3% (Fedha et al.)	Positive quality
Geldsetzer, 2016 [36]	Text message or phone call reminders for the next clinic visit vs standard of care without text or phone call reminders	Attendance at PMTCT or postnatal care clinic after delivery (**Two** studies reported this outcome for this intervention)	(*n* = 388)	**Study 1, Odeny et al*.*:** RR: 1.66, 95% CI: 1.02—2.70	Moderate quality
			(*n* = 150)	**Study 2, Kebaya et al.:** RR: 1.86, 95% CI: 1.34—2.58	
		Retention on ART at 12 months postpartum	1 (*n* = 100)	RR: 1.03, 95% CI: 0.83—1.27 (Schwartz et al.)	Very low quality

*ANC* Antenatal care, *BCG* Bacillus Calmette-Guérin vaccine, *CI* Confidence interval, *GRADE* Grading of Recommendations, Assessment, Development and Evaluations, *OR* Odd ratio, *RR* Relative risk, *VS* Versus

#### Incentive-based Interventions

One review by Till et al. [29] reported the effect of incentive-based interventions on increasing the uptake of healthcare services among pregnant women. The authors used criteria provided in the Cochrane Handbook for Systematic Reviews of Interventions [47] to assess the RoB of the included studies. The review found that the studies had low RoB. GRADE assessment for the certainty evidence was not performed (Table 4).

**Table 4:** Incentive-based interventions

Review	Interventions and Comparisons	Outcomes	Number of studies (number of participants)	Results	GRADE or Risk of Bias Assessment
Till et al., 2015 [29]	Clusters with households which received vouchers equal to cash for each pregnant woman compared to clusters which did not receive the intervention	Adequate ANC visits (≥ 5 visits)	1 (*n* = 606)	RR: 1.18, 95% CI 1.01—1.38 (Morris et al.)	Low risk of bias
		PNC visits within ten days of delivery	1 (*n* = 593)	RR: 0.43, 95% CI: 0.30—0.62 (Morris et al.)	

*ANC* Antenatal care, *CI* Confidence interval, *GRADE* Grading of Recommendations, Assessment, Development and Evaluations, *PNC* Postnatal care, *RR* Relative risk

#### Health systems: facility-based interventions

Three reviews (Tibingana-Ahimbisibwe et al. [37], Vrazo et al. [38]and Geremew et al. [39]) reported the effect of health systems facility-based interventions on increasing uptake of healthcare services among pregnant women. All three reviews did not perform GRADE assessment for the certainty of evidence. Tibingana-Ahimbisibwe et al. [37] used a validated tool from the Effective Public Health Practice Project (EPHPP) [48]. According to this tool, the review rated the study reporting outcomes relevant to this review as of moderate quality (Table 5).

**Table 5:** Health Systems – Facility-based interventions

Review	Interventions and Comparisons	Outcomes	Number of studies (number of participants)	Results	GRADE or Risk of Bias Assessment
Tibingana-Ahimbisibwe, 2015^a^ [37]	3–4 counselling and educational sessions within the facility in addition to routine prenatal care vs routine prenatal care without additional education or counselling sessions	Follow-up ANC visit	1 (*n* = 86)	Intervention: 95.3% vs Control: 16.3%, *p* < 0.001 (Mersal et al.)	Moderate quality
		Immunization uptake	1 (*n* = 86)	Intervention: 100% vs Control: 60.5%, *p* < 0.001 (Mersal et al.)	
Vrazo, 2018 [38]	Facilities with service delivery quality improvements^b^ and policy changes vs routine service delivery	Maternal ART uptake (initiation and intrapartum ART) (**Two** studies reported this outcome for this intervention)	1 (*n* = 3,552)	OR: 1.54, 95% CI: 1.29–1.85, *p* < 0.001 (Dillabaugh et al.)	Moderate quality
			1 (*n* = 1,729)	OR: 3.02, 95% CI: 2.29–3.98, *p* < 0.001 (Youngleson et al.)	
	Pre and post-intervention evaluation of integrated HIV and antenatal services in one location with one provider, laboratory courier to expedite CD4 counts, and community-based follow-up	Maternal ART uptake	1 (*n* = 624)	The proportion of ART-eligible pregnant women initiated on ART increased from 27.5% to 71.5% (RR: 2.25; 95% CI: 1.78 to 2.83; *p* < 0.01). (Herlihy et al.)	
	Facilities with Integrated ANC and ART services for pregnant women vs routine systems with separate ANC and ART locations	Maternal ART uptake(**Four** studies reported this outcome for this intervention)	(*n* = 214)	**Study 1, Turan et al.:** OR: 3.22, 95% CI: 1.67—6.23, p < 0.01	
			(*n* = 239)	**Study 2, Washington et al*.:*** OR: 2.88, 95% CI: 1.62–5.14, p < 0.01	
			(*n* = 186)	**Study 3, Weigel et al*.:*** OR 14.24, 95% CI: 6.29–32.194 (P < 0.01)	
			(*n* = 486)	**Study 4, Youngleson et al*.:*** OR: 7.07, 95% CI: 4.60–10.87 (P < 0.001)	
	Facilities with Integrated ANC and ART services for pregnant women vs routine systems with separate ANC and ART locations	Retention of mothers on ARVs during the prenatal or postpartum period (**Two** studies looked at this outcome)	(*n* = 1,172)	OR = 1.42, 95% CI: 1.11–1.83, p < 0.01 (Washington et al.)	Moderate quality
			(*n* = 166)	OR = 9.25, 95% CI: 4.14—20.6, p < 0.001 (Weigel et al.)	
Geremew, 2020 [39]	Pregnant women who had adequate antenatal care usage vs pregnant women who had no antenatal care during the index pregnancy	Postnatal care service utilization			
			14 (*n* = 21,371)	OR: 1.53, 95% CI: 1.38—1.70	

*ANC* Antenatal care, *ART* Antiretroviral therapy, *BCG* Bacillus Calmette-Guérin vaccine, *CI* Confidence interval, *GRADE* Grading of Recommendations, Assessment, Development and Evaluations, *OR* Odd ratio, *RR* Relative risk, *VS* Versus

^a^The review included 25 studies, 24 of which were from high-income settings and only one from a low-income setting. The results are from the LMIC country

^b^service delivery improvements, including rapid results delivery; increasing male partner attendance; staff redeployment and using clinic attendance data extensively to identify pregnant women who missed appointments and make follow-ups

Vrazo et al. [38] assessed the quality of each study using an eight-point rigour scale proposed by Denison et al. [49]. Overall, the included studies had low to moderate quality, with 8 (72%) studies lacking random assignment of participants to interventions, 6 (64%) studies not having equivalency between comparison groups at baseline, and none of the studies having a random selection of participants.

Geremew et al. [39] used the JBI critical appraisal tool [50] to assess the quality of the included studies. Overall, the quality of the included studies was from moderate to high, with all the studies scoring more than six points out of nine based on the appraisal tool.

#### Health systems: community-based interventions

Four reviews (Lassi et al. [40], Lassi and Bhutta [32]*,* Seward et al. [28], and Vrazo et al. [38]) assessed the effect of health systems' community-based interventions on the uptake of services among pregnant women in LMICs. The quality assessment for the review by Vrazo et al. [38] has been described in the subsection for mobile health interventions above. The reviews by Lassi et al. and Lassi and Bhutta [32, 40] did not perform GRADE assessment for the certainty of evidence. In the review by Lassi et al. [40], the authors adapted the Quality assessment tool for studies with a pre-post design proposed by Loevinsohn [51]. The authors reported that the included studies were mainly of low quality as most of them did not describe the methods of how the interventions were delivered. On the other hand, the review included one study that was of moderate quality.

In the review by Lassi and Bhutta [32], the authors assessed RoB for the included studies using the criteria outlined in the Cochrane handbook for systematic reviews [47]. The studies included in this review had a moderate RoB. Still, they scored poorly on blinding participants on the treatment they were allocated to and blinding study personnel on assessment of outcomes primarily due to the nature of the interventions. The review by Seward et al. [28] only used the GRADE criteria to assess the quality of the evidence for each outcome. The authors found that the quality of evidence for the outcome of ANC uptake was low, and the quality for facility delivery outcomes was high (Table 6).

**Table 6:** Health Systems – Community-based interventions

Review	Interventions and Comparisons	Outcome(s)	Number of studies (number of participants)	Results	GRADE or Risk of Bias Assessment
Lassi and Bhutta, 2015 [32]	Community-based interventions versus routine care^a^	Iron supplementation uptake	7 (71,622)	RR: 1.47, 95% CI: 0.99—2.17, *p* = 0.05	Moderate risk of bias
		Tetanus toxoid immunization uptake	10 (71,279)	RR: 1.05, 95% CI: 1.02—1.09, *p* < 0.01	Moderate risk of bias
		Institutional deliveries	16 (147,890)	RR: 1.2, 95% CI: 1.04—1.39, *p* = 0.01	Moderate risk of bias
Lassi et al., 2015 [40]	Training of CHWs as part of a basic package of health services	Skilled attendance at delivery (**Two** studies reported this outcome for this intervention)	NS^b^	**Study 1, Aitken et al*.*:** Skilled attendance increased from 7 to 19%	Low quality
			(*n* = 5,331)	**Study 2, Mullany et al*.*:** Skilled attendance increased from 5.1 to 48.7%	Moderate quality
		Antenatal care usage	NS^b^	**Study 1, Aitken et al*.*:** Antenatal care use increased from 8 to 32%	Low quality
	Outreach service: Safe motherhood was advocated through a mobile healthcare unit in 23 rural frontier communities. The mobile team was responsible for training community health workers and providing community education and maternal health services	Skilled attendance at delivery (**Two** studies reported this outcome for this intervention)	(n = 2,786)	**Study 1, Miranda et al*.*:** Skilled birth attendance increased significantly from 71 to 89% (p < 0.01)	
			NS	**Study 2, Wabulakombe et al.:** Skilled attendance increased from 37 to 60%	Low quality
		Antenatal care usage	NS	The antenatal consultation rate increased from 55 to 88%. (Wabulakombe et al.)	Low quality
Seward et al., 2017 [28]	**Intervention** (women’s groups with regular meetings led by a local female facilitator who received training and given training material) vs control (clusters received health service strengthening and training of traditional birth attendants without attending group meetings)	Antenatal care uptake	7 (pooled *n* = 104,797)	1. No evidence of improved antenatal care uptake (OR 1.03, 95% CI 0.77–1.38)	1. The GRADE criteria for studies for outcome 1: Low
		Institutional deliveries	6 (pooled *n* = 98,582)	2. No evidence of improved health facility delivery (OR 1.02, 95% CI 0.93–1.12)	2. The GRADE criteria for studies for outcome 2: high
Vrazo et al., 2018 [38]	Building capacity of lay healthcare providers to provide outreach PMTCT, education and support services for HIV-positive pregnant women in the community vs routine care	Maternal ART initiation and uptake	1 (*n* = 1,210)	OR: 6.39, 95% CI: 5.00—8.18, p < 0.001 (Tonwe-Gold et al.)	Moderate quality
	CHW case management in the community vs routine care	Maternal ART initiation and uptake	1 (*n* = 2,187)	OR: 10.43, 95% CI: 8.30—13.12, P < 0.001 (Kim et al.)	

*ANC* Antenatal care, *CHW* Community Health Worker, *NS* Not specified, *CI* Confidence interval, *GRADE* Grading of Recommendations, Assessment, Development and Evaluations, *OR* Odd ratio, *RR* Relative risk, *VS* Versus

^a^The community-based interventions included training CHWs to provide basic antenatal and postnatal care services in the community, conducting home visits, and convening support groups for pregnant women in their assigned catchment area

^b^Sample size not specified, but this was a country-wide project in Afghanistan between 2003 and 2006

^c^CHWs were matched with pregnant women in their catchment area to improve linkages between PMTCT, early infant diagnosis (EID) and paediatric HIV care

#### Health systems: composite interventions

One review by Brittain et al. [41] reported the effect of health-systems composite interventions on increasing the uptake of healthcare services among pregnant women. The authors did not perform quality assessments for the included studies or the certainty of the outcomes.

### Effects of interventions

#### Interventions assessing the effect of male partner involvement (Table 3)

Our search retrieved two systematic reviews (Takah et al. [33] and Suandi et al. [34]), which assessed the effect of involving male partners in improving the uptake of healthcare services among pregnant women in LMICs (Table 7). The reviews included studies that evaluated the impact of these interventions on the following outcomes: maternal ART initiation, timely ANC initiation, adequate ANC visits, receiving all components of ANC, receiving ANC services from a medically trained provider, increased institutional delivery, skilled attendance at delivery, and increased postpartum visit.

**Table 7:** Male partner involvement Interventions

Review, year	Interventions and Comparisons	Outcomes	Number of studies (number of participants)	Results	GRADE or Risk of Bias Assessment
Takah et al., 2017 [33]	Complex community intervention vs those without the intervention ^a^	Initiating maternal ART	6 (n = 8,872)	OR: 4.22, 95% CI 2.27—7.77	Moderate to low risk of bias
	Invitation letters vs no invitation letters	Initiating maternal ART	4 (*n* = 366)	OR: 1.21, 95% CI 0.89—1.63	
	Psychological counselling by trained personnel to the pregnant women together with their partners vs no counselling	Initiating maternal ART	2 (*n* = 241)	OR: 2.29, 95% CI 1.42—7.69	
	Verbal encouragement and invitation vs no verbal encouragement	Initiating maternal ART	5 (*n* = 2,015)	OR: 2.39, 95% CI 1.26—4.53	
Suandi, 2021 [34]	Pregnant women being accompanied by their male partners to ANC clinic vs standard of care with no initiative to involve male partners	Early ANC initiation (not more than 12 weeks gestation)	1 (*n* = 210)	aOR 1.05: 95% CI: 0.79—1.39 (Mohammed et al.)	- The average score for the included studies: 14/26 - External validity criteria scored lowest at 22%
		Adequate ANC visits (≥ 4 visits) (**Three** studies reported this outcome for this intervention)	(*n* = 442)	**Study 1, Mullany et al.:** OR: 1.06, 95% CI: 0.95—1.18	
			(*n* = 426)	**Study 2, Wai et al*.*:** aOR: 5.82, 95% CI: 3.34—10.15	
			(*n* = 2,642)	**Study 3, Forbes et al.:** aOR: 1.06, 95% CI: 0.82—1.38	
		Receiving recommended ANC components ^b^	1 (*n* = 2,642)	aOR: 0.65, 95% CI: 0.39—1.10 (Forbes et al.)	
		Receiving ANC services from a medically trained provider	1 (*n* = 317)	aOR: 4.50, 95% CI: 2.30—8.70 (Rahman et al.)	
	Male partner involvement in maternal health services^c^ vs standard of care with no initiative to involve male partners	Institutional delivery	6(*n* = 202,315)	pOR: 2.76, 95% CI: 1.70—4.50, *p* < 0.001	
		Skilled attendance at delivery	5 (*n* = 6,234)	pOR: 3.19, 95% CI: 1.55—6.55, *p* = 0.002	
		Post-partum visit	4 (*n* = 4,019)	pOR: 2.13, 95% CI: 1.45—3.13, *p* = 0.0001	

*aOR* Adjusted odds ratio, *ART* Antiretroviral therapy, *CI* Confidence interval, *GRADE* Grading of Recommendations, Assessment, Development and Evaluations *MD* Mean difference, *OR* Odds ratio, *pOR* Pooled: odds ratio, *RR* Relative risk, *VS* Versus

^a^The interventions involved using male champions (males considered role models in the communities) to visit HIV-positive pregnant women together with their male partners, encouraging them to initiate antiretroviral therapy (ART) including prevention to mother transmission (PMTCT) services and advising the male partner on the need to take up services. Other interventions involved community sensitization campaigns and community couple counselling by community health workers on the relevance of male partner involvement

^b^Including identification of pre-existing health conditions; early detection of complications arising during pregnancy; health promotion and disease prevention; and birth preparedness and complication planning

^C^services including the male partner attending ANC; the male partner involving the female partner in household decision-making; receiving counselling for HIV; disclosing HIV result, providing financial support, male partner being involved in birth preparedness

##### Increased maternal ART initiation

The review by Takah et al. [33] assessed the effect of four different approaches aimed at increasing male partner involvement in the prevention of mother-to-child transmission (PMTCT) of HIV infection and the impact of these approaches on the uptake of maternal ART in sub-Saharan Africa (SSA).

The first intervention utilised male champions (males considered role models in their communities) to visit HIV-positive pregnant women together with their male partners. The males champions encouraged the HIV-positive pregnant women to initiate ART and take up PMTCT services. Additionally, they provided counselling to the male partner on the need to be involved in accessing healthcare services. The interventions also involved community sensitisation campaigns and community couple counselling by community health workers on the necessity of male partner involvement in service uptake. To assess the effectiveness of this intervention, Takah et al. [27] performed a meta-analysis of six studies (one cRCT, four cohort studies, and one serial cross-sectional study). The studies were conducted in Malawi, Mozambique, Nigeria, Uganda (two studies) and Zambia, with a pooled sample size of 8,872. The review found that HIV-positive pregnant women whose male partners received the intervention were over four times more likely to initiate ART during pregnancy than those who did not (OR: 4.22, 95% CI: 2.27 – 7.77, *n* = 8,872, 6 studies).

The second intervention involved writing invitational letters to male partners of HIV-positive pregnant women to get them involved in the ART uptake of their partners. To assess this intervention, the authors performed a meta-analysis of four studies (two RCTs and two cohort studies) with a pooled sample size of 366 participants. The included studies were conducted in Malawi, Rwanda, Tanzania and Zambia. The review found that HIV-positive pregnant women whose male partners received the invitational letters were no more likely to initiate ART during pregnancy than those who did not (OR: 1.21, 95% CI: 0.89 – 1.63, *n* = 366, 4 studies).

The third intervention involved providing specialised psychological counselling by trained personnel to HIV-positive pregnant women together with their male partners. To assess the effect of this intervention, Takah et al. [27] performed a meta-analysis of two studies (one cohort study and one RCT conducted in Kenya and South Africa, respectively) with a pooled sample size of 241 participants. The review found that the HIV-positive pregnant women whose male partners received the intervention were 2.29 times more likely to initiate ART during pregnancy than those who did not receive the intervention (OR: 2.29, 95% CI: 1.42 – 7.69, *n* = 241, 2 studies).

The fourth intervention encouraged HIV-positive pregnant women verbally to bring their male partners to the ANC clinic. A meta-analysis of five cohort studies conducted in Kenya, Malawi, Nigeria, South Africa and Tanzania with a pooled sample size of 2,015 participants was conducted to assess the effect of this intervention. The review found that HIV-positive pregnant women who received verbal encouragement to bring their male partners to the ANC clinic were 2.39 times more likely to initiate ART during pregnancy than those who did not (OR: 2.39, 95% CI: 1.26—4.53, *n* = 2,015, five studies).

##### Increased adequate antenatal care visits (four or more visits)

We retrieved one systematic review by Suandi et al. [34], which investigated the effect of male partner involvement interventions on adequate ANC visits by pregnant women. The review included three studies (by Wai et al. [52], Mullany et al. [53] and Forbes et al. [54]) which assessed the effect of pregnant women being accompanied to the ANC clinic by their male partners. A cross-sectional study including 426 pregnant women conducted in Myanmar by Wai et al. [52] found that pregnant women who were accompanied to the ANC clinic by their male partners were almost six times more likely to have adequate ANC visits than those who did not (adjusted odd ratio [aOR]: 5.82, 95% CI: 3.34 – 10.15, *n* = 426). On the other hand, an RCT including 442 pregnant women conducted in Nepal by Mullany et al. [53] and secondary data analysis of the 2011 Ethiopian Demographic and Health Survey (DHS) including 2,642 pregnant women by Forbes et al. [54] found that pregnant women accompanied by their male partners were no more likely to have adequate ANC visits (OR: 1.06, 95% CI: 0.95 – 1.18, *n* = 442; and aOR: 1.06, 95% CI: 0.82 – 1.38, *n* = 2,642 respectively).

##### Increased early ANC initiation (less than 12 weeks gestation)

The systematic review by Suandi et al. [34] further investigated the effect of male partner involvement on increased early ANC initiation. The review included one cross-sectional study including 210 pregnant women conducted in Ethiopia by Mohammed et al. [55]. The study assessed the effect of the intervention involving pregnant women being accompanied to the ANC clinic by their male partners on early ANC initiation. The study found that pregnant women accompanied by their male partners were no more likely to initiate ANC early than those not (aOR: 1.05: 95% CI: 0.79 – 1.39, *n* = 210).

##### Receiving recommended ANC components

Our search retrieved one systematic review by Suandi et al. [34], which investigated the effect of male partner involvement on receiving recommended ANC components (including identification of pre-existing health conditions; early detection of complications arising during pregnancy; health promotion and disease prevention; and birth preparedness and complication planning). The review included one cross-sectional study by Forbes et al. [54], which assessed the effect of the intervention involving pregnant women being accompanied to ANC clinics by their male partners on receiving recommended ANC components. Forbes et al. [55] used the 2011 Ethiopian DHS data, which included 2,642 pregnant women. The study also found that pregnant women accompanied by their male partners were no more likely to receive recommended ANC components than those not accompanied (aOR: 0.65, 95% CI: 0.39—1.10, *n* = 2,642).

##### Receiving ANC services from a medically trained provider

The systematic review by Suandi et al. [34] included one cross-sectional study including 317 pregnant women conducted in two rural sub-districts in Bangladesh by Rahman et al. [56]. The study assessed the effect of the intervention involving pregnant women being accompanied by their male partners to the ANC clinic on receiving ANC services from a medically trained provider. The study found that pregnant women accompanied by their male partners were 4.50 times more likely to receive ANC services from a medically trained provider than those not accompanied (aOR: 4.50: 95% CI: 2.30 – 8.70, *n* = 317).

##### Increased institutional delivery

The systematic review by Suandi et al. [34] additionally included a meta-analysis which assessed the effect of interventions involving male partners in maternal healthcare services on increased institutional delivery among pregnant women in LMICs. The interventions involved: attending the ANC clinic; the male partner involving the female partner in household decision-making, receiving counselling for HIV, disclosing HIV status, encouraging the male partner to provide financial support towards the female partner’s maternal healthcare needs; and the male partner being involved in birth preparedness. The meta-analysis included six studies (one RCT, one prospective cohort study, one retrospective cohort study and three cross-sectional studies) conducted in Ethiopia, India, Malawi, Myanmar, Nepal and Zambia, with a pooled sample size of 202,315. The review found that pregnant women whose male partners were involved in maternal healthcare services were 2.76 times more likely to deliver in health facilities (pooled odds ratio [pOR]: 2.76, 95% CI: 1.70 – 4.50, *n* = 202,315, 6 studies).

##### Increased skilled attendance at delivery

The systematic review by Suandi et al. [34] also performed a meta-analysis which assessed the effect of interventions involving male partners in maternal healthcare services described in the preceding subsection on increased skilled attendance at delivery. The meta-analysis included five studies (one RCT, one prospective cohort study and three cross-sectional studies) conducted in Ethiopia (two studies), Indonesia, Kenya, and Nepal, with a pooled sample size of 6,234. The review found that pregnant women whose male partners were involved in maternal healthcare services were 3.19 times more likely to deliver in health facilities (pOR: 3.19, 95% CI: 1.55 – 6.55, *n* = 6,234, 5 studies).

##### Increased postnatal care visits

Finally, the systematic review by Suandi et al. [34] performed a meta-analysis which assessed the effect of the interventions involving male partners in maternal health care services described above on increased postnatal care visits. The meta-analysis included four studies (two RCT, one retrospective cohort study and one cross-sectional study) conducted in Burkina Faso, Myanmar, Nepal and Zambia with a pooled sample size of 4,019. The review found that pregnant women whose male partners were involved in maternal healthcare services were 2.13 times more likely to deliver in health facilities (pOR: 2.13, 95% CI: 1.45 – 3.13, *n* = 4,019, 4 studies).

### Mobile health interventions (Table 4)

Overall, our search retrieved four systematic reviews (Sondaal et al. [31], Wagnew et al. [30], Saronga et al. [35] and Geldsetzer et al. [36]) investigating the effect of mobile health interventions on the uptake of services by pregnant women in LMICs. The reviews included studies that assessed the impact of these interventions on the following outcomes: adequate ANC visits, skilled attendance at delivery, increased facility-based service utilisation, timely ANC initiation, attendance at postnatal care, retention on ART 12 months postpartum, uptake of nutritional supplements and dietary counselling (Table 3).

#### Increased adequate antenatal care visits

The review by Sondaal et al. [31] included one cRCT, which included 2,637 pregnant women, and reported that the odds of having at least four ANC visits were 2.39 times higher among pregnant women who got the mobile health intervention compared to those who did not (OR: 2.39, 95% CI: 1.03 – 5.55, *n* = 2,637).

#### Increased skilled attendance at delivery

On skilled attendance at delivery, the review by Sondaal et al. [31] found that a higher percentage of women receiving mobile health interventions had skilled attendance at delivery compared to those in the control groups (60% versus 47%, one study).

The review by Wagnew et al. [30], which included a meta-analysis of three randomised controlled trials (RCTs) with a pooled sample size of 3,282, also found that pregnant women who received mobile health interventions were more likely to have skilled attendance at delivery compared to those in control groups (pooled OR: 1.82, 95% CI: 1.33 – 2.49).

#### Other outcomes

The review by Sondaal et al. [31] reported that pregnant women who received mobile health interventions had increased facility-based service utilisation (54.5% in the intervention versus 30.6% in the control group, one study, *n* = 3,230), early ANC attendance; essential vaccines uptake (95%—100% after intervention versus 60% at baseline) and timely ANC and vaccination visits (58.68% versus 43.79%, p < 0.001 for ANC visits and 42.22% versus 34.49%, p < 0.001 for essential vaccinations).

The review by Geldsetzer et al. [36] included three RCTs which assessed the effect of text message or phone call reminders on attendance to PMTCT, postnatal care and retention into HIV care at 12 months postpartum for HIV-positive pregnant women. In two RCTs (comprising a total of 538 participants) included in the review, HIV-positive pregnant women who received the mobile health intervention were more likely to attend PMTCT or postnatal care after delivery compared to those in control groups (RR: 1.66, 95% CI: 1.02 – 2.70 and RR: 1.86, 95% CI: 1.34 – 2.58). On the other hand, one RCT, including 154 participants, did not find an association between text message reminders and retention in HIV care at 12 months postpartum (RR: 1.03, 95% CI: 0.83 – 1.27).

Two other systematic reviews (Sondaal et al. [31] and Wagnew et al. [30]) assessed the effect of mobile health interventions on adequate ANC visits (≥ 4 ANC visits before delivery). The review by Sondaal et al. [31] included a programmatic cluster randomised controlled trial (cRCT) of facilities in Tanzania and Zanzibar, including 2,550 pregnant women who reported that women in the intervention clusters were more likely to have ≥ 4 ANC visits before delivery compared to women in control clusters (OR: 2.39, 95% CI: 1.03 – 5.55). Similarly, a systematic review and meta-analysis of three RCTs by Wagnew et al. [30] found that women who received mobile health interventions were more likely to have ≥ 4 ANC visits before delivery compared to women in control clusters (OR: 2.74, 95% CI: 1.41 – 5.32).

### Incentives-based interventions (Table 5)

Our search retrieved one systematic review (by Till et al. [29]), which aimed to determine whether providing a financial incentive among pregnant women was an effective strategy to increase the uptake of services compared to the standard of care (Table 4).

#### Increased adequate ANC visits and Postnatal care (PNC) visits

The review assessed the effect of the intervention on having adequate ANC visits and PNC visits within ten days after delivery.

The review included one study from a LMIC setting, a cRCT by Morris et al. [57] The study was conducted in 70 communities with the highest malnutrition rates in rural Honduras, and included 606 pregnant women. Pregnant women in households in the intervention clusters received vouchers worth an equivalent of about £2.53 at the time every month. The cRCT reported that pregnant women in the intervention clusters were more likely to have adequate ANC visits before delivery than women in control clusters (RR: 1.18, 95% CI: 1.01 – 1.38, *n* = 606, one study). On the other hand, the cRCT reported that pregnant women who received the intervention were 57% less likely to return for PNC visits within ten days of delivery compared to women in control clusters. (RR: 0.43, 95% CI: 0.30 – 0.62, *n* = 593, one study).

### Health systems: facility-based interventions (Table 6)

Our search retrieved three systematic reviews (Tibingana-Ahimbisibwe et al. [37], Vrazo et al. [38] and Geremew et al. [39]) that included studies which assessed the effect of different facility-based health systems interventions on the uptake of services by pregnant women in LMICs. The included studies reported the impact of these interventions on the following outcomes: follow-up ANC visit and immunisation uptake; retention in ART care during prenatal and postnatal periods; maternal intrapartum ART uptake; and postnatal care service utilisation (Table 5).

#### Increased follow-up ANC visits and immunisation uptake

One systematic review by Tibingana-Ahimbisibwe et al. [37] aimed to assess the effectiveness of facility-based interventions for improving prenatal care attendance for pregnant adolescents. The review included 24 studies from high-income countries and one RCT by Mersal et al. [58] conducted in Egypt (an LMIC setting based on the World Bank criteria described in the methods section). The trial assessed the effectiveness of a facility-based program which involved providing three to four counselling and educational sessions to pregnant adolescents and routine care on increasing follow-up rates or subsequent ANC visits. The trial found that the proportion of pregnant adolescents who had follow-up visits was significantly higher (more than five times) in the intervention compared to the control group (95.3% versus 16.3%, *p* < 0.001, *n* = 86). The trial further assessed the effectiveness of the intervention on increasing essential immunisation uptake, and found that pregnant adolescents who received the additional counselling and educational sessions were more likely to receive essential vaccinations compared to those who did not (100% versus 65%, *p* < 0.001, *n* = 86).

#### Increased retention in ART care during prenatal and postnatal periods

Our search retrieved one systematic review by Vrazo et al. [38], which examined facility-based interventions for improving service uptake and retention in care among HIV positive HIV-positive pregnant women. The review included a cRCT by Washington et al. [59] and a prospective cohort study by Weigel et al. [60] conducted in Kenya and Malawi, respectively. The cRCT examined the effectiveness of integrating ANC and ART services under the same provider within the facility on retaining HIV-positive pregnant women on ART care during the prenatal and postpartum periods. Both trials found that women attending facilities with integrated ANC and ART services were more likely to be retained in care (OR: 1.42, 95% CI: 1.11 – 1.83, *p* < 0.01, *n* = 1,172; and OR: 9.25, 95% CI: 4.14 – 20.6, p < 0.01, *n* = 166).

#### Increased maternal ART uptake

The review by Vrazo et al. [38] also included two prospective cohort studies by Dillabaugh et al. [61] and Youngleson et al. [62], which assessed the effectiveness of service delivery quality improvements and changes within facilities on increasing maternal intrapartum ART initiation and uptake. The service delivery quality improvements included: rapid results delivery, increasing male partner attendance, staff redeployment and using clinic attendance data extensively to identify pregnant women who missed appointments and follow them up. Both studies found that HIV-positive pregnant women attending facilities with quality improvements were more likely to initiate ART during the intrapartum period (OR: 1.54, 95% CI: 1.29 – 1.85, p < 0.001, *n* = 3,552; and OR = 3.02, 95% CI: 2.29 – 3.98, p < 0.001, *n* = 1,729 respectively).

Furthermore, the review by Vrazo et al. [38] included a pre- and post-project evaluation from 2011 to 2013 in Zambia by Herlihy et al. [63], which assessed the effectiveness of integrating HIV and ANC services in one location by the same healthcare provider, plus expediting delivery of CD4 cell count results and community follow-up on maternal intrapartum ART initiation and uptake. Herlihy et al. [64] found that the intervention increased ART initiation among HIV-positive pregnant women from 27.5% at the start to 71.5% at the project's close.

Further, the review by Vrazo et al. included two cRCTs (by Turan et al. [64] and Washington et al. [59]) and two prospective cohort studies (by Weigel et al. [60] and Youngleson et al. [62]), which assessed the effectiveness of Integrated or increased access to ANC/ART services on maternal intrapartum ART initiation and uptake. All four studies found that women in intervention clusters were significantly more likely to initiate ART during the intrapartum period than women in control clusters (Table 5).

#### Increased postnatal care service utilisation

Our search retrieved one systematic review by Geremew et al. [39], which examined whether adequate prenatal usage (≥ 4 antenatal care visits) has a subsequent effect on postnatal care service utilisation among pregnant women. The review performed a meta-analysis of 14 RCTs with a pooled sample size of 21,371 pregnant women, which found that pregnant women who adequately utilised prenatal services were significantly more likely to use postnatal services compared to those who did not (OR: 1.53, 95% CI: 1.38 – 1.70, *n* = 21,371, 14 studies).

### Health systems: community-based interventions (Table 7)

We retrieved four systematic reviews that included studies assessing the effect of different health system's community-based interventions on the uptake of services by pregnant women in LMICs (Lassi et al. [40], Lassi and Bhutta [32], Seward et al. [28], and Vrazo et al. [38]). The systematic review included studies which reported the effect of the community-based interventions on the following outcomes: skilled attendance at delivery; increased ANC service utilisation, increased institutional delivery; iron supplementation and *tetanus toxoid* immunisation; and increased maternal ART initiation and uptake.

#### Increased skilled attendance at delivery

Our search retrieved one systematic review by Lassi et al. [40], which included studies that assessed the effect of community-based health systems interventions on increased skilled attendance at delivery. The review reported the impact of two interventions on this outcome. The first intervention was reported in a case study of the situation in Afghanistan conducted by Aitken et al. [65] and two-stage cluster-sampling pre-post surveys conducted by Mullany et al. [66] in Myanmar, which included 5,331 pregnant women. The intervention involved training lay community health workers (including community health workers, traditional birth attendants, and maternal health workers) and allowing them to work in the community. In the case study by Aitken et al. [65], skilled attendance at delivery increased from 7 to 19% at the end of the study, while in the pre-post surveys by Mullany et al. [66], skilled attendance rose from 5.1 to 48.7%.

The second intervention was also reported in pre-post surveys conducted by Miranda et al. [67] and Wabulakombe [68] in Guatemala and the Democratic Republic of Congo (DRC), respectively. The intervention advocated safe motherhood through mobile healthcare units in rural frontier communities. Teams in the mobile healthcare units provided training to community health workers on the provision of essential maternal healthcare services. The review found that in a study by Miranda et al. [67], skilled attendance at delivery rose from 71 to 89% and 37 to 60% in the surveys by Wabulakombe [68].

#### Increased ANC service utilisation

Our search retrieved two systematic reviews (Lassi et al. [40] and Seward et al. [28]), which assessed the effect of community-based health systems interventions on increased ANC service utilisation among pregnant women. The review by Lassi et al. [40] included two RCTs which further assessed the effect of the two interventions described in the above subsection on increased ANC service utilisation. The first intervention involving the training of HCWs was reported in the Afghan situation case study by Aitken et al. [65], which found that ANC service utilisation had increased from 8 to 32%.

The second intervention involving advocating safe motherhood through mobile healthcare units was reported in the pre-post survey by Wabulakombe [68] in DRC, which found that ANC service utilisation had increased from 55 to 88%.

A review and meta-analysis of cRCT by Seward et al. [28] assessed the effect of an intervention involving the utilisation of women groups on ANC uptake and facility delivery. The intervention involved conducting regular meetings for the women's groups led by a local female facilitator who had received materials and training on safe motherhood. Control clusters received training on safe motherhood for traditional birth attendants but did not have women groups. The review found no evidence of increased ANC service utilisation in the intervention groups compared to the control groups (OR: 1.03, 95% CI: 0.77 – 1.38, *n* = 104,797, 7 studies).

#### Increased institutional delivery

Our search retrieved a Cochrane review by Lassi and Bhutta [32] and a systematic review by Seward et al. [28], which assessed the effect of community-based health systems interventions on increased institutional delivery among pregnant women. Lassi and Bhutta [32] performed a meta-analysis of 16 cRCTs conducted in India, Bangladesh, Pakistan, Nepal, China, Zambia, Malawi, Tanzania, South Africa, and Ghana with a pooled sample size of 147,890 pregnant women. The analysis assessed the effectiveness of a community-based intervention which involved training of CHWs to provide essential antenatal and postnatal care services in the community, conducting home visits, and convening support groups for pregnant women in their assigned catchment areas on institutional deliveries compared to the standard of care. The review found that women in the intervention clusters were 20% more likely to deliver in facilities than women in the control cluster (RR: 1.2, 95% CI: 1.04—1.39, *p* = 0.01, *n* = 147,890, 16 studies).

The review by Seward et al. [28] performed a meta-analysis of six RCTs conducted in rural communities in Bangladesh, Malawi, and Nepal, and rural and urban communities in India with a pooled sample size of 98,582 pregnant women. The analysis assessed the intervention, which involved conducting regular women’s group meetings led by a local female facilitator who had received training and training material on safe motherhood, compared to control clusters which had received health service strengthening and training of traditional birth attendants but did not have women groups. The review did not find evidence of increased health facility delivery among pregnant women in the intervention cluster compared to women in control clusters (OR 1.02, 95% CI 0.93 – 1.12, *n* = 98,582, 6 studies).

#### Increased nutritional supplementation and immunisation uptake

The Cochrane systematic review by Lassi and Bhutta [32] further performed meta-analyses which assessed the effectiveness of the community-based intervention involving the training of CHWs to provide essential healthcare services described in the preceding subsection on increasing iron supplementation and tetanus toxoid immunisation uptake. The meta-analysis for iron supplementation uptake included seven cRCTs with a pooled sample size of 71,622 pregnant women and found that pregnant women in the intervention were 47% more likely to have iron supplementation uptake compared to pregnant women in control clusters (RR: 1.47, 95% CI: 0.99 – 2.17, *p* = 0.05, *n* = 71,622, 7 studies).

The meta-analysis for *tetanus toxoid* immunisation uptake included ten cRCTs with a pooled sample size of 71,279 pregnant women and found that pregnant women in the intervention clusters were 5% more likely to have *tetanus toxoid* immunisation uptake compared to pregnant women in control clusters (RR: 1.05, 95% CI: 1.02 – 1.09, *p* < 0.01, *n* = 71,279, 10 studies).

#### Increased maternal ART initiation and uptake

Our search retrieved one systematic review by Vrazo et al. [38], which assessed the effect of two community-based health systems interventions on increased maternal ART initiation and uptake among HIV-positive pregnant women. The first intervention was reported in a prospective cohort study including 1,210 pregnant women conducted by Tonwe-Gold et al. [69] in Cote d’Ivoire. The intervention involved building the capacity of lay healthcare providers to provide outreach PMTCT, educational, and support services for HIV-positive pregnant women in their catchment communities. The study found that women in the intervention group were more than six times more likely to initiate ART than those in the control groups (OR: 6.39, 95% CI: 5.0 – 8.18, *p* < 0.001, one study; *n* = 1,210).

The second intervention was reported in a prospective cohort study including 2,187 pregnant women conducted by Kim et al. [70] in Malawi. In the intervention groups, CHWs were matched with pregnant women in their catchment areas to improve linkages to PMTCT, early infant diagnosis (EID) and paediatric HIV care. The study found that women in the intervention group were more than ten times more likely to initiate ART than those in the control groups (OR 10.43, 95% CI: 8.30 – 13.12, *p* < 0.001, *n* = 2,187).

### Health systems: composite interventions (Table 8)

**Table 8:** Health systems – Composite interventions

Review	Interventions and Comparisons	Outcomes	Number of studies (number of participants)	Results	GRADE or Risk of Bias Assessment
Brittain et al., 2021 [41]	Pre and post evaluation study in communities that got the intervention^a^ vs communities that did not get the intervention	Early ANC initiation (< 12 weeks’ gestation) for pregnant adolescent mothers	1 (*n* = 802)	- Early ANC initiation in intervention communities increased from 8% at baseline to 56% at the end of the study, while in control communities, early ANC initiation increased from 7 to 24%. (Dyalchand et al.)	QA not done for this review
	Pregnant women aged ≥ 16 who got the intervention ^b^ vs those who did not get the intervention	Attrition from ART care six months postpartum	1 (*n* = 340)	- Attrition at six months: 19% in the intervention group vs 28% in the control group. (Fayorsey et al.)	
	Pregnant women aged ≥ 18 who got the intervention ^c^ vs those who did not get the intervention	Retention into ART care 30 days postpartum	1 (*n* = 454)	- Retention at 30 days: 92% in the intervention group vs 80% in the control group. (Mubiana-Mbewe et al.)	

*ANC* Antenatal care, *ART* Antiretroviral therapy, *CI* Confidence interval, *GRADE* Grading of Recommendations, Assessment, Development and Evaluations, *OR* Odd ratio, *QA* Quality assessment, *RR* Relative risk, *VS* Versus

^a^Monthly surveillance of adolescents’ reproductive health needs, facilitating referral to care; and providing behaviour change counselling by community health workers

^b^individual level PMTCT education; retention and adherence support; phone and SMS appointment reminders; and tracking missed clinic visits by lay counsellors

^c^Follow-up of missed visits; individual counselling; home-based couple HIV testing and counselling; male partner HIV testing; and appointment reminders by community health workers

Our search retrieved one systematic review by Brittain et al. [41], which reported the effect of three composite interventions on improving early ANC initiation (< 12 weeks gestation), reducing attrition from ART care six months postpartum, and improving retention of ART care 30 days postpartum.

#### Increased early ANC initiation

The review included one pre-post project evaluation, including 802 pregnant women, conducted by Dyalchand et al. [71] in rural India. The study assessed the effectiveness of an intervention involving monthly surveillance of adolescent reproductive health needs, facilitating referral to care and providing counselling in intervention communities by community health workers on increased early ANC initiation among pregnant adolescents. The review found that early ANC initiation increased from 8 to 56% in intervention communities compared to an increase from 7 to 24% in control communities.

#### Reduced attrition from ART care six months postpartum

The review included one RCT conducted in western Kenya by Fayorsey et al. [72] which included 340 pregnant women aged at least 16 with access to a cell phone. The RCT assessed the effect of a composite intervention on reducing attrition from ART care at six months. The intervention involved providing individual-level PMTCT education, retention adherence support, phone call and SMS reminders, and tracking of missed visits by lay counsellors. The review found that attrition from ART at six months was lower in the intervention group compared to the control group (19% vs 28%).

#### Increased retention on ART care 30 days postpartum

The review also included one RCT including 454 pregnant women conducted by Mubiana-Mbewe et al. [73] in Zambia, which assessed the effect of a composite intervention on retention into ART care at 30 days postpartum for HIV-positive pregnant women aged 18 years and older. The intervention involved follow-up of missed visits, individual counselling; home-based couple HIV testing; male partner HIV testing and appointment by community health workers. The review found that retention in the intervention group was 92% compared to 80% in the control group.

## Discussion

### Summary of main results

In this review of systematic reviews, we synthesised evidence on interventions for improving healthcare service utilization among pregnant women in LMICs. We systematically categorised the interventions into broad groups based on the outcomes measured in each systematic review. We found that mobile health interventions were effective in improving a wide range of outcomes on the maternal health continuum. In addition, interventions involving male partners were effective in improving skilled birth attendance, postnatal visits, and maternal ART uptake and retention, but were not effective in increasing the number of adequate ANC visits during pregnancy. While our results provide important insights on effective interventions, further research is needed to identify interventions for increasing early ANC initiation.

### Male partner involvement

Interventions involving pregnant women's male partners effectively increased institutional delivery (skilled birth attendance) and ART initiation during pregnancy. On the other hand, involving male partners did not increase early ANC attendance or adequate ANC visits.

We found that the reviews which included studies in which male partners were actively engaged, for example, using community male champions or providing specialised counselling, showed a positive impact of male partner involvement as these interventions potentially increased the male partners’ knowledge of pregnancy-related threats and the need to provide financial or psychological support to achieve a positive motherhood experience for their partners. On the other hand, interventions in which male partners were passively involved, like sending invitation letters to accompany their partners on the next ANC visit, did not show a positive impact.

In many LMIC settings, men are considered the primary household earners and decision-makers [34, 74, 75]. Actively involving male partners and providing them with sufficient information can increase awareness of pregnancy-related threats and provision of financial or psychological support, which would consequently result in increased healthcare service uptake among pregnant women.

### Mobile health interventions

Mobile health interventions effectively improved healthcare uptake among pregnant women on a wide range of outcomes on the maternal healthcare continuum, including timely and adequate ANC visits, skilled birth attendance and nutritional supplement uptake.

Mobile health interventions involved regular phone calls or text message reminders for subsequent appointment visits, providing psychological and moral support, and following up on missed appointments. These interventions were convenient and potentially cost-effective. As mobile phone coverage has continuously increased in LMICs [76], our results support the inclusion of mobile health interventions in improving healthcare service uptake among pregnant women.

### Incentive-based interventions

There was a paucity of evidence on the effectiveness of incentive-based interventionss. We found one systematic review which included one study from an LMIC setting which reported that a financial incentive was effective in increasing adequate ANC visits but was not effective in increasing PNC visits after the incentive was discontinued. Thus, incentives should be designed to foster continued service uptake even when the incentive is discontinued.

Though incentives can be considered an additional cost to healthcare service delivery, incentives can dramatically improve healthcare outcomes when planned well [77]. For example, in a large population-based cRCT, a small financial incentive (about US$3) was found to be a powerful motivator for increasing linkage to HIV care. The study found that the financial incentive increased linkage to HIV care among men (who generally have low service uptake [78]) by up to 51% after home-based HIV testing and referral to care [79].

### Facility-based interventions

Facility-based interventions were mainly targeted at HIV-positive pregnant women; and involved integrating ANC and ART services, expediting CD4 cell results delivery, and providing additional educational and counselling sessions. These interventions were effective in increasing subsequent ANC visits as well as maternal ART uptake and retention.

Integrating ANC and ART services with the same providers within the facility removes barriers to accessing ART services for pregnant women when the services are separated [80, 81]. Our results support the continued scaling up of ANC, ART and other forms of service integration for better service provision to pregnant women.

### Community-based interventions

Community-based interventions effectively increased immunization uptake but had mixed effects on increasing institutional delivery. We found the first intervention, which involved training CHWs to provide basic ANC and PNC services within the community, was effective. In contrast, the second intervention, in which pregnant women in intervention clusters held regular meetings led by a local facilitator who had received training, was ineffective. Within the control clusters in the second intervention, traditional birth attendants were provided safe motherhood training and had health service delivery improvements. These activities potentially increased healthcare service uptake in control clusters, thereby offsetting the gains in the intervention clusters.

Community-based interventions were further associated with a higher proportion of ANC attendance and maternal ART initiation and retention. However, the quality of evidence for these interventions was moderate to poor as the individual studies, though covering large populations, were not designed or powered to detect these effects.

### Overall completeness and applicability of evidence

We retrieved two systematic reviews assessing the effect of male involvement interventions, four systematic reviews each evaluating the impact of mobile health interventions and health systems community-based interventions, and three systematic reviews assessing the effects of health systems facility-based interventions. The reviews predominantly included studies conducted in SSA and East and South Asia. The results on the effects of these interventions are robust and mostly applicable to these settings and other geographical regions with similar cultures and demographics. The reviews predominantly reported the impact of these interventions on the increased number of subsequent ANC visits, institutional delivery, and skilled birth attendance. However, there was a paucity of evidence on interventions aimed at increasing early ANC initiation (less than 12 weeks gestation). Only two studies in two systematic reviews reported interventions aimed at improving early ANC initiation.

Additionally, there was a lack of evidence on the effect of incentive-based interventions on increasing the uptake of services among pregnant women in LMICs. We retrieved only one review by Till et al. [29], which reported the effect of incentive-based interventions. The review included five studies; among these, only one was from an LMIC setting.

### Quality of the evidence

The methodology of the systematic reviews was of high quality, with an average score of 83.1% using the AMSTAR tool. Only 3 of the 14 systematic reviews performed a GRADE assessment for the certainty of evidence. The RoB assessments showed that the included studies had low to moderate RoB. The quality of evidence for the effect of mobile health interventions on ART retention at 12 months postpartum was low. Similarly, the quality of evidence for community-based interventions was low to moderate, with high-quality evidence on the effect on institutional delivery.

### Potential biases in the review process

We developed a comprehensive search strategy. We retrieved relevant systematic reviews for this review. To reduce selection bias, we screened the retrieved records, appraised them, and extracted data in duplicate. We resolved disagreements through discussions and involving a third reviewer. Though we did not perform assessments for publication bias, we included all systematic reviews that met our inclusion criteria irrespective of whether the reviews reported positive findings or the designs of the included studies. A potential limitation of our review is that we searched only two databases (PubMed and Cochrane Library). Though these databases are widely used in the biomedical field as they are some of the major sources of systematic reviews, it is possible that some relevant reviews were missed by our search. Future reviews should expand the search to additional databases and sources.

### Authors' conclusions

#### Implications for practice

We found that the different interventions effectively improved healthcare service utilization on the maternal healthcare continuum; as such, these interventions should be implemented in combination.

Mobile health interventions should be adopted and scaled up as part of healthcare service delivery among pregnant women in LMICs.

Male partners of pregnant women should be actively engaged and be provided with sufficient information on pregnancy-related threats and their need to be involved.

ANC, ART and other forms of service integration for better service provision to pregnant women should be scaled up.

#### Implications for future research

There was a paucity of evidence on the effectiveness of incentive-based interventions. Future studies exploring cost-effective incentives which can foster healthcare service among pregnant women even when the incentive is removed are required.

We included systematic reviews, including studies with both random and non-randomised designs. Non-randomised pre- and post-project evaluations provided useful information covering large populations, which would be practically difficult to obtain in randomised studies. However, the design and reporting standards in these studies were poor. In most cases, only percentages of participants with or without the outcome were reported but not the actual number of participants. Additionally, measures of effect of the interventions were also not reported. Though these projects are not designed for scientific research purposes, improving the design and reporting standard would provide useful information covering large populations and facilitate comparisons with other studies and populations.

While the systematic reviews in this review defined adequate ANC visits as at least four, based on the previous WHO-focused ANC model [82], the new WHO guidelines recommend at least eight visits during pregnancy [5, 83]. Studies assessing this new guideline's feasibility and effect on pregnancy outcomes are required. Though this new guideline presupposes that the increased number of visits would further improve pregnancy outcomes, having an increased number of visits would be impractical for many pregnant women in rural, hard-to-reach areas. Future studies should investigate ANC models with the most significant impact on the few visits these women can make.

We found insufficient evidence of interventions aimed at increasing early ANC initiation. Future studies should explore effective interventions to improve early ANC attendance among pregnant women in LMICs.

Finally, only 3 of the 14 included reviews performed GRADE assessments for the certainty of evidence. Future systematic reviews should be conducted in a standardised manner and assess the certainty of the evidence for the reported outcomes.

### Supplementary Information

**Additional file 1: Appendix 1.** Search strategy for Medline PubMed. **Appendix 2.** Search in Cochrane library. **Appendix 3.** Data Extraction Form. **Appendix 4.** AMSTAR Tool for assessing the quality of the selected systematic reviews.

## Data Availability

All data extracted or generated during this study are included in the tables and in the supplementary material.
