# Supplementary material for: Interventions to enhance healthcare utilisation among pregnant women to reduce maternal mortality in low- and middle-income countries: a review of systematic reviews
Source: BMC Public Health. 2023 Sep 6;23:1734. doi: 10.1186/s12889-023-16558-y (PMC10481488; doi:10.1186/s12889-023-16558-y)
Supplement: Supplementary file 1 — Additional file 1: Appendix 1. Search strategy for Medline PubMed. Appendix 2. Search in Cochrane library. Appendix 3. Data Extraction Form. Appendix 4. AMSTAR Tool for assessing the quality of the selected systematic reviews. [file 12889_2023_16558_MOESM1_ESM.docx]

**Interventions to enhance healthcare utilisation among pregnant women to reduce maternal mortality in low- and middle-income countries: a review of systematic reviews**

# **Supplementary Material**

## **Appendix 1: Search strategy for Medline PubMed**

| **No** | **Search concept** | **Search terms** |
| --- | --- | --- |
| #1 | Maternal health | “Maternal health outcome*” OR “maternal health” OR gynaecology OR gynecology OR “Reproductive Health” OR “sexual health” OR “maternal welfare” OR obstetric* OR pregnanc* OR “pregnancy outcome” OR “pregnancy outcomes” OR childbearing OR “child bearing” OR prenatal OR “pre natal” OR “antenatal” OR “ante natal” OR “perinatal care” OR “peri natal care” OR “peri natal care” OR perinatal OR “peripartum* period*” OR “perinatal* period*” or “peri natal* period*” OR Parturition* OR birth OR childbirth* OR “safe delivery” OR “safely delivered” OR antepartum OR “ante partum” OR intrapartum OR “intra partum” OR Woman OR women OR Labor OR labour OR Caeserian OR Ceserean OR Birth OR “Giving birth” OR “birth giving” |
| #2 | Maternal Mortality | “Maternal mortality” OR “Maternal death” OR “Maternal fatality” OR “Maternal survival” OR death or mortality OR dead OR die OR fatality |
| #3 | Combined maternal health and mortality | #1 OR #2 |
|  | **Interventions** |  |
| #4 | Postpartum hemorrhage | “Uterine atony” OR “genital tract trauma” OR “vaginal laceration*” OR “cervical lacerations” OR “uterine rupture” OR “prolonged labour” OR “prolonged labor” OR anaemia OR anemia OR “Postpartum hemorrhage” OR “Postpartum haemorrhage” OR “third stage of labor” OR “third stage of labour” OR “blood loss” OR Bleeding OR “excessive bleeding” OR “uncontrolled bleeding” OR “blood transfusion” OR “blood bank” OR uterotonic OR “prophylactic uterotonic*” OR oxytocin OR “oral oxytocin” OR “injectable oxytocin” OR “ergot alkaloid*” OR “Prophylactic oxytocin” OR ergometrine OR carbetocin OR ergometrine OR methylergometrine OR “injectable prostaglandin*” OR prostaglandin* OR “Uterine massage” OR “early cord clamping” OR “cord clamping” OR “controlled cord traction” OR “cord traction” |
| #5 | Preeclampsia and eclampsia | Eclampsia OR Preeclampsia OR “pre eclampsia” OR “pre-eclampsia” OR “Hypertensive disorder*” OR “pregnancy hypertension” OR “pregnancy hypertensive disorder*” OR “generalized seizure*” OR seizure* OR aspirin OR “low dose aspirin” OR “low-dose aspirin” OR “blood pressure” OR “Monitor blood pressure” OR “blood pressure Monitor” “blood pressure control*” OR “control blood pressure” OR “controlling blood pressure” OR “urinalysis” |
| #6 | Maternal sepsis | Sepsis OR “maternal sepsis” OR “genital tract sepsis” OR “puerperal fever” OR “puerperal sepsis” OR “puerperal infection” OR “pelvic pain” OR “pelvis pain” OR fever OR “abnormal vaginal discharge” OR “abnormal discharge” OR “abnormal smell” OR “foul odour” OR “foul odor” OR “uterine involution” OR “group B Streptococcus colonization” OR “Streptococcus colonization” OR “rupture* membrane*” OR “vaginal examination” OR “vaginal cleansing” OR “routine antibiotic prophylaxis” OR “antibiotic prophylaxis” OR “prophylactic antibiotic” OR ampicillin OR gentamicin OR clindamycin |
| #7 | Obstructed labour | “Obstructed labour” OR “obstructed labor” OR dystocia OR “Breech Presentation” OR breech OR “breech position” OR “abnormal labor” OR “abnormal labour” OR “delayed labor” OR “delayed labour” OR “Uterine Hemorrhage” OR “vaginal haemorrhage” OR “vaginal hemorrhage” OR “vaginal bleeding” |
| #8 | Skilled birth attendance | “Skilled attendan*” OR “skilled birth attendan*” OR “traditional birth attendan*” OR “traditional attendan*” OR “birth attendant” OR “mid wife” OR midwife OR midwives OR midwifery |
| #9 | Nutritional interventions | “Vitamin * supplement*” OR “vitamin supplement* “ OR “mineral supplement*” OR “nutrition* supplement*” OR “protein supplement*” OR “balanced protein supplement*” OR “energy supplement*” OR “iron supplement*” OR “iron folate supplement*” OR “folic supplement*” OR “folic acid supplement*” OR “calcium supplement*” OR “Nutrition* intervention*” OR “Nutrition treatment” OR “Nutrition Management” OR “malnutrition management” OR “malnutrition treatment” OR Malnutrition OR “Mal nutrition” OR undernutrition OR “under nutrition” OR “Food fortification” OR “Complementary feeding” OR “iodine supplement*” OR iodis* OR “salt iodis*” OR “zinc supplement*” |
| #10 | Treating infections | “Treat infection*” OR “monitor infection*” OR “Treat disease*” OR “monitor disease*” OR "HIV"[Mesh] OR "acquired immunodeficiency syndrome"[MeSH Terms] OR Hiv OR “Human immunodeficiency virus” OR aids OR “acquired immunodeficiency syndrome” OR HIV infection OR HIV positive OR HIV seropositive OR seropositive OR HIV infected OR “Sexually Transmitted Disease*” OR “Sexually Transmitted infection*” OR chlamydia OR syphilis OR gonorrhea OR gonorrhoea OR wart* “anal wart*” OR “genital wart*” OR herpes OR “genital herpes” OR “anal herpes” OR ARV OR ART OR antiretroviral therapy OR antiviral drug* OR “Option B plus” OR antiretroviral* OR malaria OR “plasmodium falciparum” OR “sulfadoxine pyrimethamine” OR “bed net*” OR “treated net” OR “treated bed net” OR “insecticide treated bed net*” OR “insecticide treated net*” OR antibiotic OR ceftriaxone OR erythromycin OR metronidazole OR penicillin OR “benzathine penicillin” |
| #11 | Safe abortion | Abort OR abortion OR “safe abortion” OR “legal abortion” OR “therapeutic abort*” OR “induced abortion” OR “pregnancy termination” OR “safe pregnancy termination” OR “legal pregnancy termination” OR “miscarriage” OR “miscarriage management” OR Misoprostol OR misoprostil OR Mifepristone OR “vacuum aspiration” |
| #12 | Planned pregnancy | “Birth Intervals” OR “birth spacing" OR “birth spacings” OR “child spacing” OR “family planning” OR “pregnancy interval” OR “safe motherhood” OR “Sexual Abstinence” OR celibacy or “postpartum abstinence” or “sexual abstinence” OR “birth regulation” OR “birth control*” or “fertility control” OR “fertility method” OR “fertility control method*” OR “contraception” OR “contraception method*” OR “conception control” OR “Emergency Contraception Pill” OR “Morning After Pill” OR contraceptive* OR “contraceptive method*” OR “Contraceptive Injection” OR injection OR “injectable contraceptive*” OR progestogen OR progesterone OR “Contraceptive Device” OR “Contraceptive Implant” OR implant OR implants OR “Intrauterine Device” OR “intra uterine device*” OR “cervical cap” OR “intracervical device” OR “Lippes loop” OR “vaginal diaphragm” OR “vaginal ring” OR “tubal ligation” OR patch OR Condom OR “Female condom*” OR “Male condom*” |
| #13 | Delayed sex debut | “teen sex” OR “teenage sex” OR “teen pregnancy” OR “adolescent sex” OR “adolescence sex” OR “adolescent pregnancy” OR “adolescence pregnancy” OR “Delayed sex” OR “sex debut” OR “sexual debut” OR “early sexual debut” OR “unprotected intercourse” OR “adolescent sex education” OR “sex education” OR “youth friendly health services” OR “youth friendly” OR “adolescent-friendly” OR “adolescent friendly” OR “Teen* Pregnancy Prevent*” OR “sex instruction” |
| 14 | Women empowerment interventions | “Intimate Abuse” OR “Partner Abuse” OR “Spouse Abuse” “Spousal Abuse” OR “Intimate partner Abuse” OR “physical abuse” OR “wife abuse” OR “gender-based abuse” OR “gender-based abuse” OR “gender based abuse” OR “sexual abuse” OR “domestic Abuse” OR “Partner rape” OR “Spousal rape” OR “wife rape” OR “Intimate assault” OR “Partner assault” OR “Spouse assault” OR “Spouse assault” OR “Intimate partner assault” OR “physical assault” OR “wife assault” OR “sexual assault” OR “domestic assault” OR abuse OR rape OR violence OR “sexual violence” OR exploit OR exploitation OR “sexual exploitation” OR force* OR harass* OR offend |
| #15 | Combined interventions | #4 OR #5 OR #6 OR #7 OR #8 OR #9 OR #10 OR #11 OR #12 OR #13 OR #14 |
|  | **Low- and middle-income countries** | |
| #16 | Low- and middle-income countries | “Low and middle income country” OR “Low and middle income countries” OR “low income” OR “lower income” OR “Low income country” OR “Low income countries” OR “middle income country” OR “middle income countries” OR “lower middle income” OR “lower middle income country” OR “lower middle income countries” OR “poor country” OR “poor countries” |
| #17 | Regions: | Africa OR “Sub-Saharan Africa” OR “sub-Sahara” OR "Africa South of the Sahara"[Mesh] OR “North Africa” OR “middle east*” OR “East Asia” OR “Central Asia” OR “South Asia” OR “Latin America” OR “South America” OR “Caribbean” OR Pacific |
| #18 | Countries | Afghanistan OR Algeria OR Angola OR Bangladesh OR Belize OR Benin OR Bhutan OR Bolivia OR Burkina Faso OR Burundi OR “Cabo Verde” OR Cape Verde OR Cambodia OR Cameroon OR “Central African Republic” OR Chad OR Comoros OR “Comoros Island” OR “Democratic Republic of Congo” OR Congo OR “Republic of Congo” OR “Côte d'Ivoire” OR “Ivory Coast” OR Djibouti OR Egypt OR “Arab Republic of Egypt” OR El Salvador OR Eritrea OR Eswatini OR Swaziland OR “The Kingdom of Eswatini” OR Ethiopia OR “The Gambia” OR Gambia OR Ghana OR Guinea OR Guinea-Bissau OR Haiti OR Honduras OR India OR Indonesia OR Iran OR “Islamic Republic of Iran” OR Kenya OR Kiribati OR “North Korea” OR “Democratic People's Republic of Korea” OR “Kyrgyz Republic” OR Kyrgyzstan OR “Lao PDR” OR Laos OR “Lao People's Democratic Republic” OR Lesotho OR Liberia OR Madagascar OR Malawi OR Mali OR Mauritania OR Micronesia OR Mongolia OR Morocco OR Mozambique OR Myanmar OR Nepal OR Nicaragua OR Niger OR Nigeria OR Pakistan OR “Papua New Guinea” OR Philippines OR Rwanda OR Samoa OR “São Tomé and Principe” OR Senegal OR “Sierra Leone” OR “Solomon Islands” OR Somalia OR “South Sudan” OR Sri Lanka OR Sudan OR “Syrian Arab Republic” OR Syria OR Tajikistan OR Tanzania OR Timor-Leste OR Togo OR Tunisia OR Uganda OR Ukraine OR Uzbekistan OR Vanuatu OR Vietnam OR “West Bank” OR Gaza OR Yemen OR “Yemen Republic” OR Zambia OR Zimbabwe |
| #19 | LMIC Combined | #16 OR #17 OR #18 |
| #20 | Combined searches | #3 AND #15 AND #19 |
| #21 | Period: 2000 – 2021 | Filter [#20]: period |
| #22 | Systematic Reviews | Filter [#21]: systematic reviews and Meta-analyses |

## **Appendix 2: Search in Cochrane library**

| **No** | **Search concept** | **Search terms** |
| --- | --- | --- |
| #1 | **Maternal health**  [Mesh term search] | Maternal health/ |
| #2 | **Maternal health**  [title and abstract phrase search] | “Maternal health outcome*” OR “maternal health” OR gynaecology OR gynecology OR “Reproductive Health” OR “sexual health” OR “maternal welfare” OR obstetric* OR pregnanc* OR “pregnancy outcome” OR “pregnancy outcomes” OR childbearing OR “child bearing” OR prenatal OR “pre natal” OR “antenatal” OR “ante natal” OR “perinatal care” OR “peri natal care” OR “peri natal care” OR perinatal OR “peripartum* period*” OR “perinatal* period*” or “peri natal* period*” OR Parturition* OR birth OR childbirth* OR “safe delivery” OR “safely delivered” OR antepartum OR “ante partum” OR intrapartum OR “intra partum” OR Woman OR women OR Labor OR labour OR Caeserian OR Ceserean OR Birth OR “Giving birth” OR “birth giving” |
| #3 | Maternal health combined | #1 OR #2 |
| #4 | **Maternal mortality**  [Mesh term search] | Maternal mortality/ |
| #5 | **Maternal Mortality**  [Title and abstract phase search] | ((Maternal) NEXT (mortality OR death OR fatality OR survival)) death or mortality OR dead OR die OR fatality |
| #6 | Combined maternal mortality search | #4 OR #5 |
| #7 | Combined maternal health and maternal mortality | #3 AND #6 |
|  | **Interventions** |  |
| #8 | **Postpartum hemorrhage**  [Title and abstract phase search] | “Uterine atony” OR “genital tract trauma” OR “vaginal laceration*” OR “cervical lacerations” OR “uterine rupture” OR “prolonged labour” OR “prolonged labor” OR anaemia OR anemia OR “Postpartum hemorrhage” OR “Postpartum haemorrhage” OR “third stage of labor” OR “third stage of labour” OR “blood loss” OR Bleeding OR “excessive bleeding” OR “uncontrolled bleeding” OR “blood transfusion” OR “blood bank” OR uterotonic OR “prophylactic uterotonic*” OR oxytocin OR “oral oxytocin” OR “injectable oxytocin” OR “ergot alkaloid*” OR “Prophylactic oxytocin” OR ergometrine OR carbetocin OR ergometrine OR methylergometrine OR “injectable prostaglandin*” OR prostaglandin* OR “Uterine massage” OR “early cord clamping” OR “cord clamping” OR “controlled cord traction” OR “cord traction” |
| #9 | **Preeclampsia and eclampsia**  [Title and abstract phase search] | Eclampsia OR Preeclampsia OR “pre eclampsia” OR “pre-eclampsia” OR “Hypertensive disorder*” OR “pregnancy hypertension” OR “pregnancy hypertensive disorder*” OR “generalized seizure*” OR seizure* OR aspirin OR “low dose aspirin” OR “low-dose aspirin” OR “blood pressure” OR “Monitor blood pressure” OR “blood pressure Monitor” “blood pressure control*” OR “control blood pressure” OR “controlling blood pressure” OR “urinalysis” |
| #10 | **Maternal sepsis**  [Title and abstract phase search] | Sepsis OR “maternal sepsis” OR “genital tract sepsis” OR “puerperal fever” OR “puerperal sepsis” OR “puerperal infection” OR “pelvic pain” OR “pelvis pain” OR fever OR “abnormal vaginal discharge” OR “abnormal discharge” OR “abnormal smell” OR “foul odour” OR “foul odor” OR “uterine involution” OR “group B Streptococcus colonization” OR “Streptococcus colonization” OR “rupture* membrane*” OR “vaginal examination” OR “vaginal cleansing” OR “routine antibiotic prophylaxis” OR “antibiotic prophylaxis” OR “prophylactic antibiotic” OR ampicillin OR gentamicin OR clindamycin |
| #11 | **Obstructed labour**  [Title and abstract phase search] | “Obstructed labour” OR “obstructed labor” OR dystocia OR “Breech Presentation” OR breech OR “breech position” OR “abnormal labor” OR “abnormal labour” OR “delayed labor” OR “delayed labour” OR “Uterine Hemorrhage” OR “vaginal haemorrhage” OR “vaginal hemorrhage” OR “vaginal bleeding” |
| #12 | **Skilled birth attendance**  [Title and abstract phase search] | “Skilled attendan*” OR “skilled birth attendan*” OR “traditional birth attendan*” OR “traditional attendan*” OR “birth attendant” OR “mid wife” OR midwife OR midwives OR midwifery |
| #13 | **Nutritional interventions**  [Title and abstract phase search] | “Vitamin * supplement*” OR “vitamin supplement* “ OR “mineral supplement*” OR “nutrition* supplement*” OR “protein supplement*” OR “balanced protein supplement*” OR “energy supplement*” OR “iron supplement*” OR “iron folate supplement*” OR “folic supplement*” OR “folic acid supplement*” OR “calcium supplement*” OR “Nutrition* intervention*” OR “Nutrition treatment” OR “Nutrition Management” OR “malnutrition management” OR “malnutrition treatment” OR Malnutrition OR “Mal nutrition” OR undernutrition OR “under nutrition” OR “Food fortification” OR “Complementary feeding” OR “iodine supplement*” OR iodis* OR “salt iodis*” OR “zinc supplement*” |
| #14 | **Treating infections**  [Title and abstract phase search] | “Treat infection*” OR “monitor infection*” OR “Treat disease*” OR “monitor disease*” OR HIV OR “Human immunodeficiency virus” OR aids OR “acquired immunodeficiency syndrome” OR HIV infection OR HIV positive OR HIV seropositive OR seropositive OR HIV infected OR “Sexually Transmitted Disease*” OR “Sexually Transmitted infection*” OR chlamydia OR syphilis OR gonorrhea OR gonorrhoea OR wart* “anal wart*” OR “genital wart*” OR herpes OR “genital herpes” OR “anal herpes” OR ARV OR ART OR antiretroviral therapy OR antiviral drug* OR “Option B plus” OR antiretroviral* OR malaria OR “plasmodium falciparum” OR “sulfadoxine pyrimethamine” OR “bed net*” OR “treated net” OR “treated bed net” OR “insecticide treated bed net*” OR “insecticide treated net*” OR antibiotic OR ceftriaxone OR erythromycin OR metronidazole OR penicillin OR “benzathine penicillin” |
| #15 | **Safe abortion**  [Title and abstract phase search] | Abort OR abortion OR “safe abortion” OR “legal abortion” OR “therapeutic abort*” OR “induced abortion” OR “pregnancy termination” OR “safe pregnancy termination” OR “legal pregnancy termination” OR “miscarriage” OR “miscarriage management” OR Misoprostol OR misoprostil OR Mifepristone OR “vacuum aspiration” |
| #16 | **Planned pregnancy**  [Title and abstract phase search] | “Birth Intervals” OR “birth spacing” OR “birth spacings” OR “child spacing” OR “family planning” OR “pregnancy interval” OR “safe motherhood” OR “Sexual Abstinence” OR celibacy or “postpartum abstinence” or “sexual abstinence” OR “birth regulation” OR “birth control*” or “fertility control” OR “fertility method” OR “fertility control method*” OR contraception OR “contraception method*” OR “conception control” OR “Emergency Contraception Pill” OR “Morning After Pill” OR contraceptive* OR “contraceptive method*” OR “Contraceptive Injection” OR injection OR “injectable contraceptive*” OR progestogen OR progesterone OR “Contraceptive Device” OR “Contraceptive Implant” OR implant OR implants OR “Intrauterine Device” OR “intra uterine device*” OR “cervical cap” OR “intracervical device” OR “Lippes loop” OR “vaginal diaphragm” OR “vaginal ring” OR “tubal ligation” OR patch OR Condom OR “Female condom*” OR “Male condom*” |
| #17 | **Delayed sex debut**  [Title and abstract phase search] | “teen sex” OR “teenage sex” OR “teen pregnancy” OR “adolescent sex” OR “adolescence sex” OR “adolescent pregnancy” OR “adolescence pregnancy” OR “Delayed sex” OR “sex debut” OR “sexual debut” OR “early sexual debut” OR “unprotected intercourse” OR “adolescent sex education” OR “sex education” OR “youth friendly health services” OR “youth friendly” OR “adolescent-friendly” OR “adolescent friendly” OR “Teen* Pregnancy Prevent*” OR “sex instruction” |
| #18 | **Women empowerment interventions**  [Title and abstract phase search] | “Intimate Abuse” OR “Partner Abuse” OR “Spouse Abuse” “Spousal Abuse” OR “Intimate partner Abuse” OR “physical abuse” OR “wife abuse” OR “gender-based abuse” OR “gender-based abuse” OR “gender based abuse” OR “sexual abuse” OR “domestic Abuse” OR “Partner rape” OR “Spousal rape” OR “wife rape” OR “Intimate assault” OR “Partner assault” OR “Spouse assault” OR “Spouse assault” OR “Intimate partner assault” OR “physical assault” OR “wife assault” OR “sexual assault” OR “domestic assault” OR abuse OR rape OR violence OR “sexual violence” OR exploit OR exploitation OR “sexual exploitation” OR force* OR harass* OR offend |
| #19 | **Combined interventions**  [Title and abstract phase search] | #8 OR #9 OR #10 OR #11 OR #12 OR #13 OR #14 OR #15 OR #16 OR #17 OR #18 |
|  | **Low- and middle-income countries** | |
| #20 | **Sub-Saharan Africa**  [Mesh term search] | Africa South of the Sahara |
| #21 | **Low- and middle-income countries**  [Title and abstract phase search] | “Low and middle income country” OR “Low and middle income countries” OR “low income” OR “lower income” OR “Low income country” OR “Low income countries” OR “middle income country” OR “middle income countries” OR “lower middle income” OR “lower middle income country” OR “lower middle income countries” OR “poor country” OR “poor countries” |
| #22 | **Regions:**  [Title and abstract phase search] | Africa OR “Sub-Saharan Africa” OR “sub-Sahara” OR “North Africa” OR “middle east*” OR “East Asia” OR “Central Asia” OR “South Asia” OR “Latin America” OR “South America” OR “Caribbean” OR Pacific |
| #23 | **Countries**  [Title and abstract phase search] | Afghanistan OR Algeria OR Angola OR Bangladesh OR Belize OR Benin OR Bhutan OR Bolivia OR Burkina Faso OR Burundi OR “Cabo Verde” OR Cape Verde OR Cambodia OR Cameroon OR “Central African Republic” OR Chad OR Comoros OR “Comoros Island” OR “Democratic Republic of Congo” OR Congo OR “Republic of Congo” OR “Côte d'Ivoire” OR “Ivory Coast” OR Djibouti OR Egypt OR “Arab Republic of Egypt” OR El Salvador OR Eritrea OR Eswatini OR Swaziland OR “The Kingdom of Eswatini” OR Ethiopia OR “The Gambia” OR Gambia OR Ghana OR Guinea OR Guinea-Bissau OR Haiti OR Honduras OR India OR Indonesia OR Iran OR “Islamic Republic of Iran” OR Kenya OR Kiribati OR “North Korea” OR “Democratic People's Republic of Korea” OR “Kyrgyz Republic” OR Kyrgyzstan OR “Lao PDR” OR Laos OR “Lao People's Democratic Republic” OR Lesotho OR Liberia OR Madagascar OR Malawi OR Mali OR Mauritania OR Micronesia OR Mongolia OR Morocco OR Mozambique OR Myanmar OR Nepal OR Nicaragua OR Niger OR Nigeria OR Pakistan OR “Papua New Guinea” OR Philippines OR Rwanda OR Samoa OR “São Tomé and Principe” OR Senegal OR “Sierra Leone” OR “Solomon Islands” OR Somalia OR “South Sudan” OR Sri Lanka OR Sudan OR “Syrian Arab Republic” OR Syria OR Tajikistan OR Tanzania OR Timor-Leste OR Togo OR Tunisia OR Uganda OR Ukraine OR Uzbekistan OR Vanuatu OR Vietnam OR “West Bank” OR Gaza OR Yemen OR “Yemen Republic” OR Zambia OR Zimbabwe |
| #24 | LMIC Combined | #20 OR #21 OR #22 OR #23 |
| #25 | Combined searches | #7 AND #19 AND #24 |
| #26 | Period: 2000 – 2021 | Filter [20]: period |
| #27 | Systematic Reviews | Filter [21]: systematic reviews and Meta-analyses |

## **Appendix 3: Data Extraction Form:**

| Reviewer ___________________________ | Review Date _______________________ | |
| --- | --- | --- |
| Author _____________________________ | Year _________ | Record number _____ |

| **1** | **Description of Systematic review** | | |
| --- | --- | --- | --- |
|  | a | First author |  |
|  | b | Title |  |
|  | c | year of publication, |  |
|  | d | Aim of the review (Review question/objective) |  |

| **2** | **Search strategy** | | |
| --- | --- | --- | --- |
|  | a | Date of search |  |
|  | b | Search period (Dates covered) |  |
|  | c | Number of databases (the databases) |  |
|  | d | Supplementary searches |  |

| **3** | **Inclusion and Exclusion criteria** | | |
| --- | --- | --- | --- |
|  | a | Inclusion criteria |  |
|  | b | Exclusion criteria |  |
|  | c | Number of Included studies (number of participants included) |  |
|  | d | Location (country) of included studies |  |
|  | e | Designs of included studies |  |
|  | f | Characteristics of included participants (age, etc.) |  |
|  | g | Relevant outcomes (the definition used in the review) |  |
|  | h | GRADE assessment of relevant outcomes |  |
|  | i | Method used to assess the risk of bias and summary |  |
|  | j | Author reported conflict of interest |  |

| **4** | **Description of Intervention** | | |
| --- | --- | --- | --- |
|  | a | Intervention |  |
|  | b | Type of intervention (Educational/Prevention/treatment etc.) |  |
|  | c | Mode of delivery of intervention |  |
|  | d | Dose (mean range) or composition or form of application (including compound, formulation) |  |
|  | e | Frequency of delivery of intervention |  |
|  | f | Start of intervention or duration (or both) |  |
|  | g | Adherence to the intervention |  |
|  | h | Any comments |  |

| **5** | **Effect of intervention** | | |
| --- | --- | --- | --- |
|  | a | Outcome |  |
|  | b | Comparator |  |
|  | c | Results (Risk/odds ratios, 95% CIs or narratives) |  |
|  | d | Number of studies reporting the outcome and Sample size of pooled data |  |
|  | e | GRADE Assessment |  |
|  | f | Summary findings |  |
|  | g | Any comments |  |

## **Appendix 4: Assessment of quality for selected reviews**

Criteria for AMSTAR:

| Reviewer ___________________________ | Date _______________________ | |
| --- | --- | --- |
| Author _____________________________ | Year _________ | Record number _____ |

|  |  | Yes | No |
| --- | --- | --- | --- |
| 1 | A priori design provided |  |  |
| 2 | Duplicate study selection and data extraction |  |  |
| 3 | Comprehensive literature search performed |  |  |
| 4 | Status of publication used as an inclusion criterion |  |  |
| 5 | List of studies (included and excluded) provided |  |  |
| 6 | Characteristics of included studies provided |  |  |
| 7 | Quality of included studies assessed and documented |  |  |
| 8 | Quality of included studies used appropriately in formulating conclusions |  |  |
| 9 | Appropriate methods used to combine the findings of the studies |  |  |
| 10 | Likelihood of publication bias assessed |  |  |
| 11 | Conflict of interest stated |  |  |
|  | Total score |  |  |
